# Adaptive Potential and Genomic Vulnerability of Keystone Forest Tree Species to Climate Change: A Case Study in Scots Pine

**DOI:** 10.1111/eva.70180

**Published:** 2025-12-05

**Authors:** Bartosz Łabiszak, Witold Wachowiak

**Affiliations:** ^1^ Department of Plant Ecology and Environmental Protection, Institute of Environmental Biology Adam Mickiewicz University in Poznań Poznań Poland; ^2^ Department of Genetics and Environmental Interactions Institute of Dendrology, Polish Academy of Sciences Kórnik Poland

**Keywords:** assisted migration, forest management, genomic offset, local adaptation, resilience strategies

## Abstract

A better understanding of the possible adaptive response and genomic vulnerability of forest trees is needed to properly assist future forest management and develop adequate resilience strategies to changing environments. Scots pine ( *Pinus sylvestris* L.), a keystone species with extensive distribution and a broad ecological niche, is expected to be directly impacted by climate change due to maladaptation and associated fitness declines. Despite extensive studies that have clarified the broad‐scale history and genetic structure of the species, understanding the genetic basis for local adaptation and the extent of genomic offset in Scots pine remains incomplete. Here, we used thousands of genotyped SNP markers in 39 natural populations (440 trees) along a broad latitudinal gradient of species distribution to examine molecular signatures of local adaptation. Specifically, this landscape genomics approach aimed to assess fine‐scale patterns of SNPs associated with environmental gradients, estimate genomic offset as a proxy for exposure and sensitivity components of vulnerability, and evaluate the adaptive response of populations to projected climate shifts. The variation of outlier SNPs, which exhibit selection signatures between genetically very similar populations in the analysed distribution range, was highly correlated with mean annual temperature, a key limiting factor for the growth and survival of tree species. Furthermore, our simulation results indicated a high genomic offset on a large spatial scale in *P. sylvestris* , with the time frame required to close the offset gap by natural selection estimated to be in the range of hundreds of years. We evaluate the genomic offset in the coming decades and indicate the optimal allelic frequency spectra required in the future to ensure resilience of Scots pine populations. We discuss forest assisted migration (FAM) as a management strategy, involving the relocation of genotypes to areas with matching environmental conditions. By evaluating adaptive responses, the study adds to the discussion on the long‐term sustainability of forest ecosystems.

## 1 Introduction

Many keystone temperate forest tree species have a large distribution range and occupy habitats across steep climatic gradients. In response to varying selection pressures across both temporal (e.g., climatic fluctuations) and spatial (heterogeneous environments) scales they not only maintain a high level of background genetic variation (orders of magnitude higher than in other perennial plants), but also show considerable levels of phenotypic plasticity and exhibit signatures of local adaptation due to natural selection, despite extensive gene flow (Hamrick and Godt 1996; Hamrick 2004; Nybom 2004; Petit and Hampe 2006; Franks et al. 2014). These mechanisms likely evolved to ensure persistence in the face of environmental upheavals and are of crucial importance in long‐lived sessile organisms that lack behavioral responses to changing environments (Alberto et al. 2013). Consequently, from a short‐term perspective, the adaptive potential of trees is almost entirely reliant on standing genetic variation. Studies have shown that in rapidly changing environments, populations adapt more effectively when beneficial alleles are part of the standing genetic variation (Thurman and Barrett 2016; Barrett and Schluter 2008; Aitken et al. 2008). However, how this adaptive potential translates into resilience under different magnitudes of environmental change remains difficult to quantify.

Increasing pressure on forest ecosystems due to environmental change raises questions about tree conditions and performance considering their specific life history traits (longevity, long generation time), complex demographic histories, and population structures. Some levels of resilience to large‐scale environmental perturbations are already known for those species, as glacial cycles during the Pleistocene shaped their genetic structure, especially in the Northern Hemisphere (Hamrick 2004; Alberto et al. 2013; Davis and Shaw 2001). However, the magnitude and tempo of current climatic shifts are unprecedented in the historical record (Intergovernmental Panel on Climate Change 2023), and many studies predict that substantial shifts in distribution ranges of species might be required to follow the optimal climatic envelopes (Dyderski et al. 2018; Pecchi et al. 2019; Thuiller 2005). Another possible way to cope with increasing selective pressure due to a rapidly changing environment is the adaptive response of populations, but still less is known about the underlying genetic architecture and potential, confounding factors (i.e., plastic response) and limits of such adaptive response (Franks et al. 2014; Nogués‐Bravo et al. 2018). Therefore, a better understanding of the link between the geographical distribution of adaptive genetic variation and its molecular signatures, along with the associations between genomes and the environment, is essential. These insights could inform decisions regarding the management of both economically important commercial forests and natural forests of great ecological value, that will face increased pressure from environmental changes.

Scots pine ( *Pinus sylvestris* L.) is an outcrossing and wind‐pollinated species that naturally forms random mating populations across extensive areas of Europe and Asia. Given its wide distribution range and significant commercial importance, Scots pine stands are one of the most ecologically and socially valuable forest trees across Eurasia (Pyhärjärvi et al. 2020). The genetic variation observed in *P. sylvestris* has been shaped by several interrelated factors, including changes in population ranges and sizes during glacial periods, gene flow between populations, and natural selection responding to adaptation to local environmental conditions (Savolainen et al. 2007). Numerous molecular genetic studies have provided insights into the broad‐scale history of Scots pine populations in Europe and Asia, contributing to their current geographic range and the observed phenotypic variability established during and after the species' postglacial recolonization (Soranzo et al. 2000; Cheddadi et al. 2006; Semerikov 2018). These studies have revealed the spatial structure of several genetic groups within the species (Naydenov et al. 2007; Naydenov et al. 2011; Pyhärjärvi et al. 2007; Dering et al. 2017; Wachowiak et al. 2023; Bruxaux et al. 2024) and the persistence of a distinctive genetic lineage, despite putative gene flow across large geographical areas (Łabiszak and Wachowiak 2023). This research provided a comprehensive understanding of standing natural variation within Scots pine. However, much less is known about the adaptive potential of this species across its range and consequently, the relative climate vulnerability of its populations (Hall et al. 2021).

In the face of ongoing environmental changes, it is particularly important to consider the possibility of adaptive responses of populations affected by shifting climatic conditions as shown across various study systems (Filipe et al. 2022; Tigano et al. 2023; Tigano et al. 2020; Markova et al. 2023; Sang et al. 2022; Koo et al. 2017). Species distribution models under various climate change scenarios indicate that many forest tree species will need to shift their geographical distribution to align with their ecological niche, but some evidence suggests that many species may not be able to keep pace with the rapid rate of climate change (Aitken et al. 2008; Dyderski et al. 2018; Zhu et al. 2012; Sittaro et al. 2017; Iverson and McKenzie 2013). The massive decline of coniferous forests in the temperate zone of Europe is predicted by distribution models (Dyderski et al. 2018; Hanewinkel et al. 2013; Schueler et al. 2014). Scots pine, in particular, might suffer not only from the direct effects of climate change on its distribution but also from increased competition with deciduous species such as oaks and beeches. The predicted mismatch between suitable climatic niches and the genetic composition of species will likely impact silviculture and forestry on a large scale, requiring the appropriate management plans that should accommodate changing climates (Oliver et al. 2016). Assisted migration might be needed to counter these challenges (Aitken and Whitlock 2013). However, such approaches would require revision of existing rules and guidelines on the origin of reproductive materials at national scales.

Estimating the adaptive potential of Scots pine across its range is crucial to evaluate the climate vulnerability of wild populations and identify those that might exhibit greater resilience to changing environments. In the most recent IPCC framework, vulnerability is defined as the propensity or predisposition to be adversely affected by climate‐related hazards, and is shaped by sensitivity and adaptive capacity (Intergovernmental Panel on Climate Change 2023). In biodiversity assessments, it is also emphasized that vulnerability integrates exposure, sensitivity, and adaptive capacity, with the latter including phenotypic plasticity, evolutionary potential, and dispersal ability (Foden et al. 2019). Our analyses focus on genomic offset, defined as the predicted allele‐frequency shifts required to maintain current genotype–environment relationships under changing climatic conditions. Genomic offset therefore reflects the exposure and sensitivity components of vulnerability but does not account for adaptive capacity and can be understood as a relative indicator of potential maladaptation under future climates. This information can guide strategies for enhancing forest management and conservation, ensuring the continued health of socially and economically significant forests. Here, we utilize data from thousands of polymorphic loci located in the coding regions of *P. sylvestris* sampled across an environmental gradient in Europe, combined with environmental data to (1) identify genomic regions correlated with the environmental factors driving local adaptation; (2) calculate the genomic offset for each population to predict climate change vulnerability; and (3) assess the extent and timeframe of the adaptive response to predicted climate, considering the species' biology. Finally, based on our results, we discuss the impact of genomic offset on managing genetic resources for forestry in the face of ongoing environmental change.

## 2 Material and Methods

### 2.1 Plant Material and Sampling

We sampled 39 populations of Scots pine spanning a broad latitudinal transect from northern Finland to southern Poland (latitudes 49.1°N–69.8°N) thereby capturing much of the species' north–south climatic variation in Europe (Figure S1A,B; Table S1). Those include populations from northern Finland (FIN1‐21; FIN) to southern Poland (POL1‐15; POL) and three populations from the Baltic Region (LVA, LTU, EST). For each population needles were collected from 5 to 23 (median = 10) randomly selected mature trees that were at least 50 m apart. In total, we analyzed 440 individuals.

### 2.2 SNP Genotyping and Data Processing

Genomic DNA was extracted from needles using a Genomic Mini AX Plant kit following the manufacturer's instructions (A&A Biotechnology, Poland). The quantity of DNA was measured by Qubit 4 fluorometer, using the Broad Range (BR) Assay Kit and DNA was diluted to the working concentration of 40 ng/μL. We acquired the single nucleotide polymorphism (SNP) genotypes of all individuals using a custom Axiom PineGAP SNP array (Affymetrix, Thermo Fisher Scientific, Santa Clara, CA, USA) as described in (Perry et al. 2020). Briefly, the development of the array followed identification of SNPs through targeted sequencing of candidate genes and transcriptomes from pine species, including Scots pine and representing samples from a broad species distribution in Europe (Perry et al. 2020). A final set of 49,829 SNP probes was selected based on their quality and allele frequency in the discovery panel to include in most cases a single SNP per gene. The subset of those SNPs was also checked to represent genomic regions distributed across different linkage groups in Scots pine (Kastally et al. 2022). The processing of the array was carried out at the Bristol Genomics Facility (Bristol, UK). Genotypes were called using Axiom Analysis Suite software (Applied Biosystems, Waltham, MA, USA), following the manufacturer's recommendations. We changed the default quality filters to more stringent, adjusting the sample threshold configuration parameters as follows: QC call_rate was set to 95%, Average cut‐rate for passing samples to 95%, and Cr‐cutoff to 95%. Those parameters control genotyping quality and SNP filtering. QC call_rate sets the minimum per‐sample call rate, ensuring only high‐quality samples are retained. Average cut‐rate defines the acceptable mean call rate across probes to filter out low‐quality SNPs. cr‐cutoff applies a SNP‐level threshold, removing variants with insufficient call rates. Initially, our set of SNPs was composed of genotypes assigned to BestAndRecommended (52.79%) category by Axiom Analysis Suite software, and we exported those SNPs to the ped file format. Then, several filtering steps were performed to extract high‐confidence, informative and polymorphic SNPs in PLINK v.1.07 (Purcell et al. 2007). First, we removed 93 SNPs with BLAST hits to *Pinus taeda* mitochondrial genome (NC_039746.1) and *Pinus sylvestris* chloroplast genome (NC_035069.1). Next, SNPs with allele frequency (MAF) < 0.05, and loci/individuals with more than 10% missing data were discarded. This conservative MAF filter was applied to reduce the influence of very rare alleles (e.g., singletons), which can introduce noise into population structure inference (Linck and Battey 2019). After the quality control steps we obtained the set of 10,597 SNPs that was used in all subsequent analyses, with the remaining missing genotypes mean‐imputed within populations for landscape genomic analyses to ensure complete data matrices. The overall proportion of missing data was very small (mean number of missing genotypes per individual = 0.03) and was randomly distributed, so this approach is unlikely to affect downstream results. We additionally conducted linkage disequilibrium (LD) pruning to remove SNPs that were highly linked (LD *r* ^2^ > 0.7) and used this subset of 6995 SNPs only in population structure analysis (PCA, LEA)—as those methods might be biased by the presence of non‐independent markers.

### 2.3 Population Structure and Genetic Diversity

We calculated observed (*H*_*o*_) and expected heterozygosity (*H*_*e*_) and fixation index *F*_IS_ in each population and overall using the *hierfstat* package in R (Goudet 2005; R Core Team 2025). Genetic differentiation among populations was determined using the pairwise *F*_ST_ calculated according to Weir and Cockerham (Weir and Cockerham 1984) in *adegenet* (Jombart and Ahmed 2011; Jombart 2008). Population genetic structure was inferred using the *LEA* R package (Frichot and François 2015; Frichot et al. 2014) and the function snmf that computes least‐squares estimates of ancestry proportions and ancestral allelic frequencies and evaluates the quality of fit of the statistical model to the data by using a cross‐validation technique (Frichot and François 2015). We tested for the number of ancestral clusters (*K*) ranging from *K* = 1 to *K* = 40, using 10 replications for each *K* to determine cross‐entropy. The results were visualized using the POPHELPER Structure Web App v1.0.10 (Francis 2017). Population‐level ancestry coefficients were calculated by a custom R script and visualized on a map using *ggplot2* (Wickham 2016; Wickham 2011). The distribution of genetic variation was also assessed by principal component analysis (PCA) in the *adegenet* R package. We first performed PCA utilizing the complete dataset and all of the sampled populations to reveal potential population subdivisions, elucidating range‐wide patterns of genetic variation. Next, we looked at PCAs within each region (excluding populations from Latvia, Lithuania and Estonia). This localized approach was aimed at unraveling the fine‐scale genetic structure patterns and potentially uncovering genetic signatures unique to each region's ecological context that might have remained concealed within the broader PCA. Finally, to assess the impact of outlier SNPs on the identified genetic structure, we performed supplementary PCA analyses encompassing two scenarios: one including the full dataset of SNPs and another excluding the SNPs found to be outliers (see Section 2.4. Outlier Detection for details).

To understand the interplay of genetic differentiation, geographical distances, and environmental factors, we employed a Mantel test approach. First, we tested the correlation between population genetic distances (as linearized pairwise *F*_ST_ values; *F*_ST_/(1—*F*_ST_)) and geodistances to search for patterns of isolation by distance (IBD). Geographic coordinates were transformed into Vincenty's geodesic that correctly accounts for the curvature of the Earth using the distVincentyEllipsoid function from the *geosphere* package (Hijmans 2022), and Euclidean distances were calculated using the dist function before performing the Mantel test in *vegan* with 999 permutations to assess statistical significance (Oksanen et al. 2024). Similarly, we performed the Mantel test on environmental distances to determine the degree of isolation by environment (IBE). Environmental distances were calculated as Mahalanobis distances using the D2.dist function from the *biotools* package (da Silva 2021) based on the first two PCs from PCA analysis performed on a set of eight selected variables (see Section 2.4 Outlier Detection section for details on variable selection). While Mantel tests have known statistical limitations, particularly in cases of complex spatial dependence and autocorrelation (Guillot and Rousset 2013; Legendre et al. 2015), they remain a useful exploratory tool when used alongside more sophisticated modeling approaches. Given that our primary analyses rely on genome‐environment association methods, the Mantel test serves as a supplementary assessment of general patterns of genetic and environmental structuring.

### 2.4 Outlier Detection

We conducted outlier detection analyses using three different methods: *F*_ST_ outlier scan, detection based on population structure, and genotype‐environment association (GEA) method. First, we used OutFLANK v0.2 (Whitlock and Lotterhos 2015; Whitlock and Lotterhos 2014) to compute a trimmed distribution of *F*_ST_ values consisting of the supposedly selectively neutral markers (not subjected to selection) and then, compared this to the *F*_ST_ distribution of all loci in the dataset. Then, using the *qvalue* R package (Storey et al. 2022) we performed the false discovery rate (FDR) correction using a *q*‐value threshold of 0.05 to control for multiple testing, ensuring that the false positive rate is minimized, to identify loci that have significantly high or low *F*_ST_ values that cannot be explained by selectively neutral factors alone and thus are likely subjected to selection.

Next, we used the *pcadapt* v4.3.3 R package (Privé et al. 2020) to detect outliers that are related to population structure using robust Mahalanobis distance. In this method, the population structure is first ascertained with PCA, and then Cattell's graphical rule is used to choose the number of principal components (K) to identify SNPs involved in local adaptation. Compared to other outlier detection methods, *pcadapt* produces fewer false positive results and is less computationally demanding (Luu et al. 2017). When using methods that utilize only genomic data for outlier detection (*OutFLANK* and *pcadapt*; hereafter we refer to those as genomic outlier scans) we followed the same procedure using all populations, representing a full environmental gradient and separately for populations from Poland (POL) and Finland (FIN) to track both large and fine‐scale patterns of local adaptation in Scots pine.

Finally, to detect linear relationships between genetic variations and multivariate environmental variables, allowing us to link outlier loci with environmental conditions we used redundancy analyses (RDAs) in *vegan*. Population‐level allele frequencies were used as the dependent matrix. We ran both full RDAs (with environment, geography, and both combined as predictors) and partial RDA (pRDA). The geographic distance matrix used in RDA was distance‐based Moran's eigenvector maps (dbMEMs) which represent a spatial decomposition of distances between populations. This framework allowed us to estimate the independent and shared contributions of environment and geography to genetic variation. Model significance was evaluated using the anova.cca function in *vegan* and 999 permutations. For these analyses, we used three genotype matrices: (i) all SNPs, (ii) SNPs identified as outliers by both *pcadapt* and *OutFLANK*, and (iii) SNPs consistently identified across all three approaches (*pcadapt*, *OutFLANK*, and RDA), to compare how SNP panel choice affects variance partitioning. To properly represent environmental variation at species' distribution we analyzed 25 environmental variables (e.g., temperature, precipitation, soil pH, and phenological indices; see Table S2 for a list of all). After reducing multicollinearity (Dormann et al. 2013), we retained eight variables with correlation coefficient |*r*| < 0.7 and Variance Inflation Factors (VIF) < 10: annual mean temperature (mean_temp), mean diurnal range (mean_dr), temperature seasonality (temp_s), precipitation of driest month (perc_dry_m), precipitation of wettest quarter (perc_wet_q), topsoil pH (ph), number of days receiving ≥ 0.1 mm precipitation (wet), and organic carbon content (carbon) using pairs. panels and vif.cca functions in *vegan* (Zuur et al. 2010) (Table S2). The geographic distance matrix was created from 10 dbMEMs retained after the forward selection step in the *adespatial* R package (*p* < 0.05), from the initial set of 38 variables (Dray et al. 2024).

Additionally, we used RDA to detect outlier loci. Specifically, to determine which SNPs could be assigned as SNPs with putative adaptive variation, we used a cutoff point of ±2.5 standard deviations (a common statistical criterion for outlier detection that has also been applied in landscape genomics e.g., de la Torre 2021) from the distribution of individual SNP loadings in the ordination space for the constrained axes explaining most of the variance. The significance of constrained axes was first assessed visually using a scree plot and then verified by permutation tests (anova.cca, by = “axis”, 999 permutations). To gain insight into the function of those variants potentially affected by selection, we conducted a BLAST analysis using transcriptomic regions containing the focal SNPs as queries (Wachowiak et al. 2015). The sequences were searched against the NCBI nucleotide database to identify homologous regions, and accession numbers were recorded for all significant matches. When available, gene names and predicted functions were extracted from the database to provide functional annotations.

### 2.5 Genomic Offset Assessment

To minimize false positives and retain a robust set of loci putatively under selection, we considered only SNPs detected by all three outlier methods (*pcadapt*, *OutFLANK*, and RDA) as potential adaptive variants (PAV), which were subsequently used in the genomic offset analyses. We then examined their associations with environmental predictors, focusing on variables showing the strongest correlations with PAV in the RDA analysis (see *Results*). Next, we applied a generalized linear model (GLM) using a binomial regression with a logit link function, to estimate the probability (*p*) of observing a particular genotype at a given locus as a function of the environmental predictor (mean_temp): log(p/(1–*p*)) = β₀ + β₁ × temperature + ε, where *p* represents the probability that a given genotype (minor homozygote, major homozygote) is present at a locus, β₀ is the intercept, β₁ is the regression coefficient for the environmental variable, and ε is the residual error. The model allowed us to quantify the strength and direction of association between the frequencies of SNP genotypes and environmental variation. Statistical significance was assessed using the *p*‐value, and Z‐scores, indicating whether the association between genotype occurrence and the predictor variable is significantly different from zero. The goal of this approach was to explicitly model the probability of genotype occurrence across environmental gradients.

Next, to evaluate the potential selective challenges that Scots pine populations may face under future climate scenarios, we used three complementary approaches to assess genomic offset: (1) RONA–RDA, an RDA‐based adaptation of the Risk of Nonadaptedness framework, which quantifies the mean absolute change in allele frequencies (Δp) necessary for populations to remain optimally adapted to future projected environmental conditions (Rellstab et al. 2016; Bay 2018); (2) RDA offset, which estimates the Euclidean distance between adaptive indices derived from redundancy analysis under present and future climates following (Capblancq and Forester 2021); and (3) Gradient Forest (GF), a non‐linear machine learning approach that models multilocus allele–environment associations using regression trees and projects them into an importance‐weighted environmental space (Ellis et al. 2012; Gougherty et al. 2021). We decided to check the accuracy of predictions of those three methods, comparing the simpler RONA–RDA method, which offers a much more intuitive interpretation of the genomic offset score (being simply the amount of shift in allele frequencies), to more sophisticated methods, which were shown recently to provide more accurate predictions of genomic offset (Lind and Lotterhos 2025). While all three approaches quantify potential genomic responses to environmental change, RONA–RDA focuses on locus‐specific allele frequency shifts, whereas RDA offset and GF capture broader, multivariate genomic–environment relationships. Additionally, to evaluate the impact of SNP set choice on genomic offset predictions and to test the robustness of our conclusions across alternative marker panels we applied them to two sets of SNPs: one consisting of putatively adaptive variants concordant across all outlier‐detection methods (PAVs), and another consisting of variants identified as outliers by at least two independent methods.

The baseline climatic variables were downloaded from WorldClim v2.1 (Fick and Hijmans 2017) for the 1970–2000 reference period, while non‐climatic predictors (e.g., soil properties, UV‐B, frost days) were obtained from publicly available datasets (see Table S2). For each sampling location, we then extracted future bioclimatic variables (2050; average for years 2041–2060) according to the climatic model BCC‐CSM2‐MR (Wu et al. 2019), projected under three Shared Socioeconomic Pathway (SSP) scenarios: SSP 126, SSP 245, and SSP 585 from WorldClim v2.1 at a spatial resolution of 30 s (~1 km^2^). These pathways represent future conditions ranging from relatively mild to severe climatic change (SSP 126 < SSP 245 < SSP 585). Non‐climatic predictors were retained at their present values, as future projections are not available. Because the RDA signal was dominated by mean annual temperature (see Results), offset patterns primarily reflect climatic gradients, and retaining these predictors preserved consistency with the baseline genotype–environment associations.

For RONA‐RDA based genomic offset, we calculated the average absolute change in allele frequencies (Δp). This metric was calculated following the original formula 1/n Σ₍ᵢ₌₁₎ⁿ |pᵢ—pᵢ*|, where pᵢ represents the observed frequency of allele i, pᵢ* is the optimal frequency predicted from genotype–environment associations for that allele (we refer hereafter to this change in allele frequency as ∆*p* (Rellstab et al. 2016; Bay 2018)). We contrasted current allele frequencies per population for PAV with allele frequencies projected under future environmental conditions, calculated using the *AlleleShift* R package for all PAVs and each SCP scenario (Kindt 2021). Despite the strong correlation between allele frequencies and mean annual temperature, as well as the observed linear relationship with this environmental variable (see *Results*), we avoided using simple linear regression. Instead, we used *AlleleShift*, which first calibrates a redundancy analysis (RDA) model to establish baseline allele‐environment relationships, followed by a generalized additive model (GAM) with a binomial family to predict future allele frequencies, ensuring values remain within biologically meaningful bounds (0–1). Unlike the original RONA implementation (Rellstab et al. 2016), which employed generalized linear models, our RONA–RDA approach derives predicted allele frequencies from multivariate RDA loadings. The genomic offset score was then calculated for each population as the mean difference between the observed current and future expected allele frequencies, and the extrapolated values of genomic offset were visualised for the studied transect using the Inverse Distance Weighting (IDW) implemented in the *gstat* R package (Gräler et al. 2016). When using RDA‐offset analysis, adaptive indices were calculated for current and future climates, and genomic offset was defined as the Euclidean distance between these positions. We then projected the genomic offset score on a map, for our entire study transect, separately for each climatic scenario, and then extracted genomic offset scores for each population. Similarly, for GF‐based we estimated the multivariate environmental distance between present and future climates, weighted by the importance of allele–environment associations inferred from regression trees following the implementation described by Gougherty et al. (2021), who used GF to estimate genomic offset and climate maladaptation in a forest tree species. As with RDA, we projected GF offsets across the transect for each scenario and provided population‐level values of genomic offset. For figure comparability across all methods and scenarios, we visualised genomic offset values as indexed scores (10 classes) for direct comparison. We then compared the general spatial patterns and examined the correlations among estimates of genomic offset across all methods.

To illustrate the timeframe for populations to follow the predicted changes in allele frequencies in future environmental conditions, we calculated the number of generations required for a genetic shift of such magnitude, with natural selection being the sole driver of this change. Because we found high concordance between patterns produced by both genomic offset assessments (see *Results*), we used RONA‐RDA scores to calculate the baseline timeframe of changes in allele frequencies that will match required in novel climate. We simulated a simple scenario, based on the basic population genetic formula for the change in allele frequency in one generation given selection: ∆*p* = (sp_0_q_0_^2^)/(1—s *q_0_^2^), where the change in allele frequency is only a function of the differing strength of the selection coefficient, and all SNPs are dominant (*h* = 0). We set the change of allele frequency (∆*p*) to be equal to the maximum and minimum values of genomic offset score under SSP 585, and we simulated scenarios with different strengths of the selection coefficients (in range: 0.1–0.9). We deliberately did not account for dominance, ignored effective population size, and gene flow, as we were only interested in a lower bound estimate of time—and adding those parameters into our model will only prolong the time required to change allele frequencies. We transformed the time from a number of generations into calendar years by assuming an average generation time of 20–25 years in *P. sylvestris* (Boratyński 1993; Wachowiak et al. 2011). All simulation and data visualization were done using a custom‐written R script.

## 3 Results

### 3.1 Genetic Diversity and Patterns of Population Structure

**FIGURE 1:**
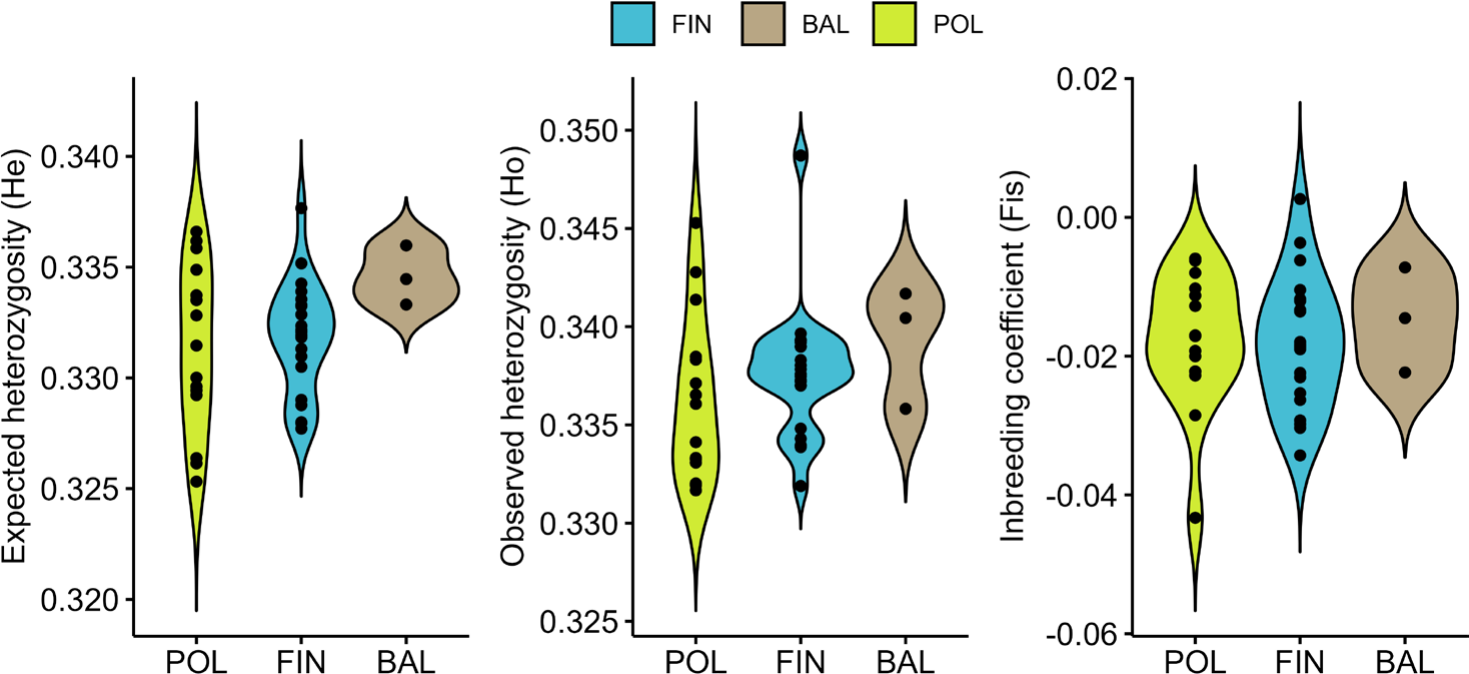
Genetic diversity on a regional scale in Scots pine. The violin plots show expected heterozygosity (*H*_*e*_), observed heterozygosity (*H*_*o*_), and fixation index (*F*_IS_) within three regions—Finland (FIN), Baltic Region (BAL) and Poland (POL). See Table S3 for basic descriptive statistics at the population level and the assignment to regions.

The overall level of observed heterozygosity (*H*_*o*_) was 0.3372, while expected heterozygosity (*H*_*e*_) was 0.3319. The diversity levels recorded for individual populations were found to be similar across the studied transect and within the regions (Figure 1, Table S3). Following the high outbreeding rate of this species, no indication of inbreeding was found in our dataset, as the values of the *F*_IS_ index were close to 0 (Figure 1, Table S3). Pairwise *F*_ST_ across all populations was low with a global estimate of 0.017. The divergence was higher between Poland and Finland (*F*_ST_ = 0.021), and within regions, the *F*_ST_ was almost twofold higher in Poland when compared to Finland (0.017 and 0.010, respectively). This result was mainly due to populations POL2, POL6, POL14 and POL15 which showed the highest values across all pairwise comparisons, leading to significantly elevated levels of *F*_ST_ among populations in Poland, where it was comparable to *F*_ST_ recorded between geographical regions (Figure S2). The Mantel test results showed that despite the low level of divergence between populations, an increase in *F*_ST_ was statistically significantly correlated with both geographic and environmental distances between populations (Figure S3). However, the strength of those correlations differed and indicated that IBD is stronger than IBE (*R* ^2^ = 0.19 vs. 0.11, respectively, both with *p* < 0.001, Figure S3A,B).

**FIGURE 2:**
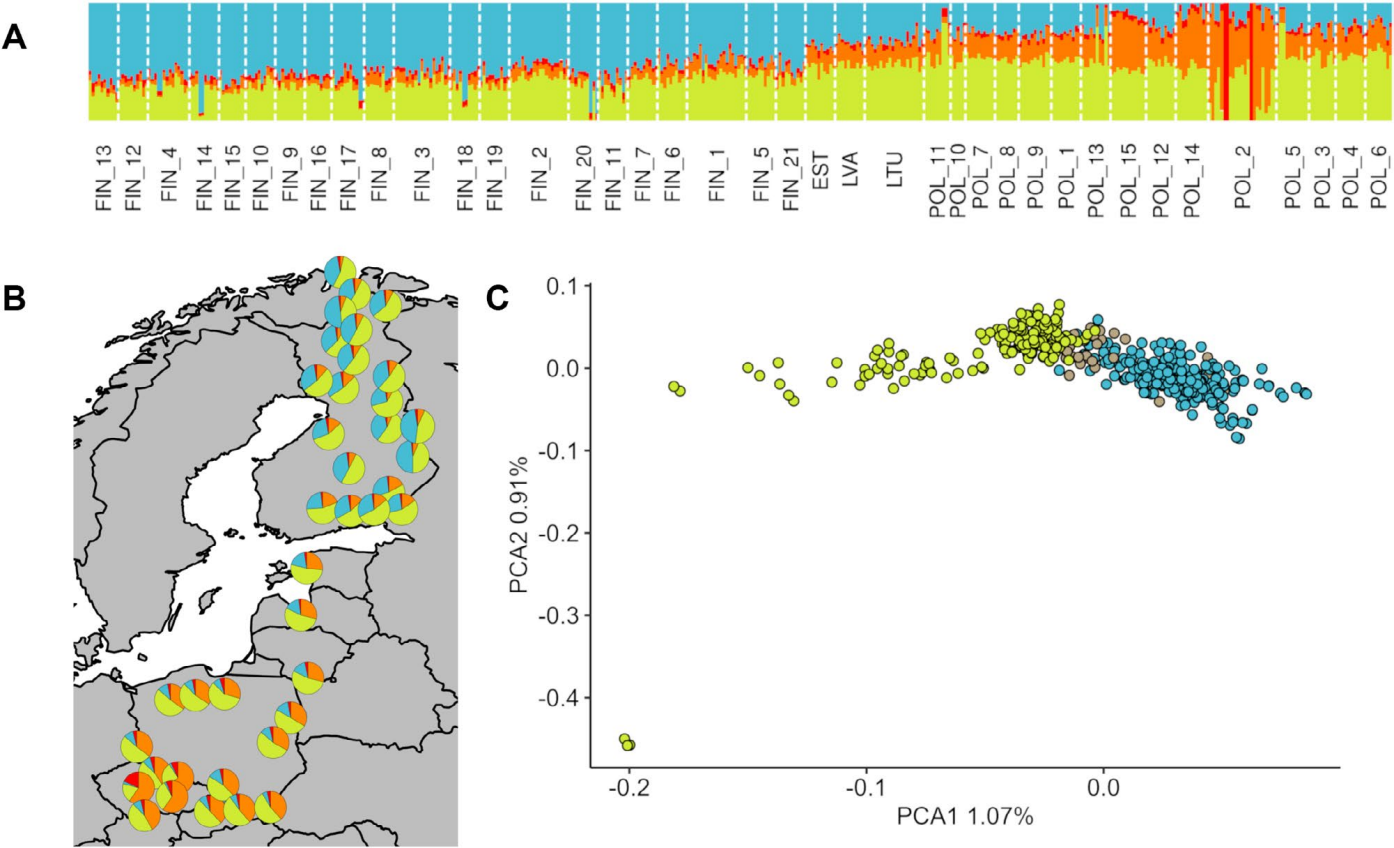
Scots pine population structure across latitude. (A) The proportion of ancestry in each individual tree in 39 populations (sampling sites) inferred using LEA for *K* = 4 indicated as the most likely number of clusters (*K*) or ancestral lineages by the cross‐entropy criterion (see Figures S4 and S5 for results of cross‐entropy and *K* values ranging from 2 to 10). Four different colors of the bars representing individual trees correspond to the four suggested clusters or ancestral lineages. Populations (sampling sites) are ordered from north to south. (B) Geographic distribution of the ancestry coefficients at the population level with *K* = 4. (C) Principal component analysis (PCA) projections of all 440 trees of *P. sylvestris* . Individual trees are consistently color‐coded according to their geographic origin: Finland—light blue, Baltic Region—grey, Poland—lime green.

Genetic structure analysis using LEA showed a cline‐like clustering pattern strongly associated with latitude but separating Polish from Finnish populations (Figure 2A,B). The cross‐entropy criterion showed two significant drops at *K* = 4 and *K* = 10; however, increasing the number of clusters (*K*) above four did not indicate any additional substructure, but rather reflected the pattern already present at *K* = 4, so we continue to use this number (Figures S4 and S5). The first cluster represented mostly ancestry or lineage of Finnish populations (highlighted by the blue colour in the LEA plot in Figure 2). It is decreasing substantially from north to south, and in Poland it contributes less than 20% to the genome (per population). In contrast, the Polish populations are characterised by a higher ancestry proportion of two clusters (highlighted by lime green and orange colours in the LEA plot in Figure 2, respectively), the latter (orange) being very rare in the north. Interestingly, the population from Stołowe Mts. (POL2, POL13‐15) shows not only the highest proportion of this ancestry, but some trees from this region are so diverged that they form their own cluster (highlighted by red colour in the LEA plot in Figure 2). PCA performed with LD‐pruned SNPs (6995) showed a consistent pattern with the LEA results of population structure driven by geography (Figure 2C). We also looked at the fine‐scale patterns of population structure within regions, but found only a weak pattern in Finland and Poland. In Finland, we saw to some extent the structure between the north and the south, while in Poland the most distinctive were populations from Stołowe Mountains, which is consistent with earlier analyses (FigureS6A,B). To determine to what extent outlier loci (potentially under selection) influence population structure, we performed PCA using three datasets: the full SNP set (6995 SNPs), the full set excluding 20 PAVs (6975 SNPs) and a dataset containing only PAVs (Figure S7). Interestingly, excluding PAVs did not change the overall population structure pattern (Figure S7C), while when we used only outlier loci in PCA we recovered a similar pattern but with lower discriminating power (Figure S7C,D). No such pattern was present, when we randomly selected the same number of SNPs as outlier loci and ran 10,000 permutations (see Figure S7, for details).

### 3.2 Genetic‐Environmental Associations

**FIGURE 3:**
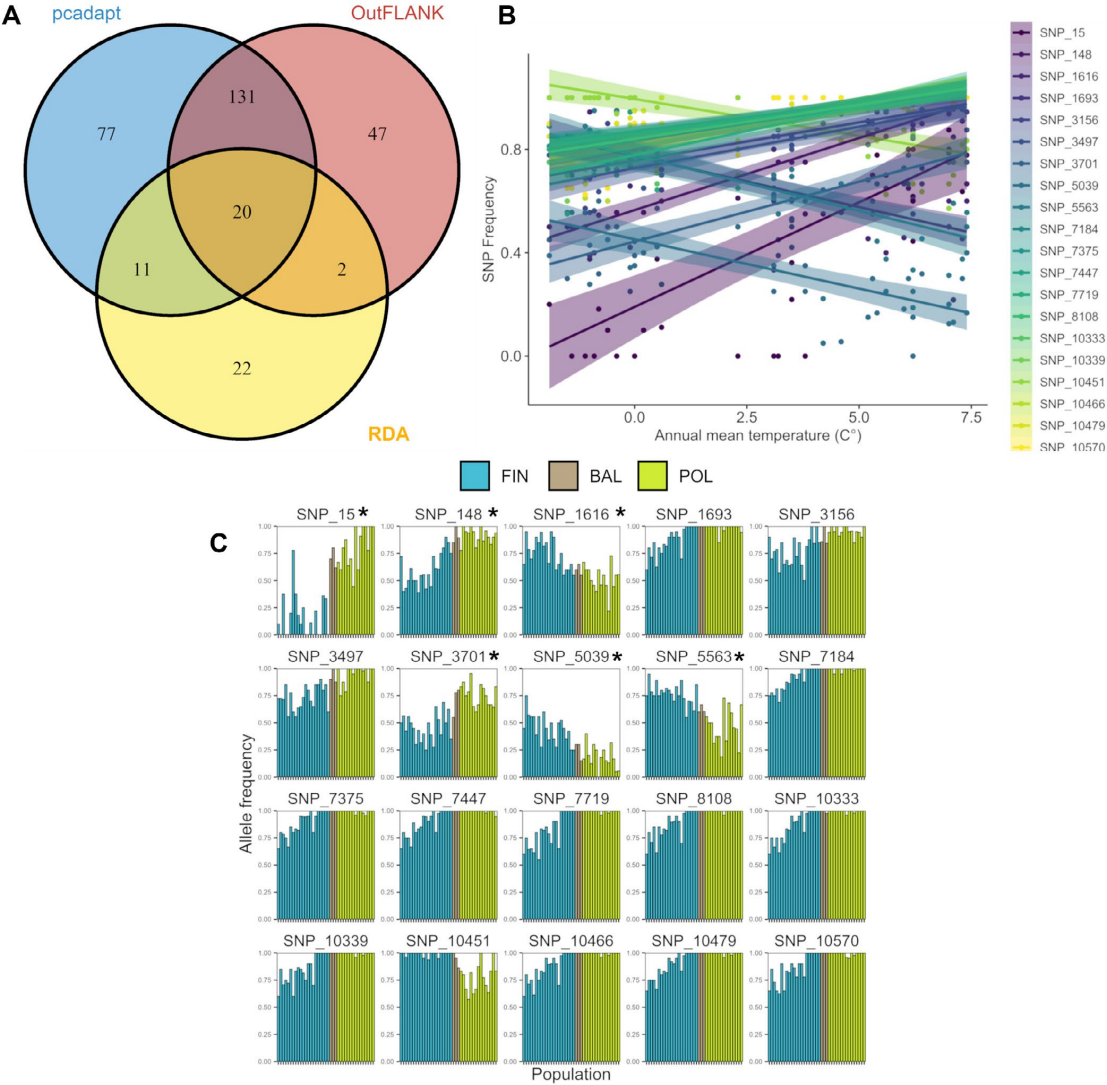
Candidate adaptive loci detected in *P. sylvestris* . (A) Venn diagram with the number of SNPs detected as outliers by three different methods. Only 20 SNPs that are consistent among all those methods were selected as potential adaptive variants (PAV). (B) Linear relationships of 20 PAVs and the most related environmental variable. The points indicate the position of the different populations, with the solid line and the highlighted region showing the linear relationship between the environment and the frequency (shaded area = 95% confidence interval). Each line/shading and colour indicates the relationship of a single SNP with the mean annual temperature. (C) Bar plots representing allele frequencies of PAV showing the clinal pattern within the studied populations. The sites are ordered from north to south, and populations are colour‐coded as in Figure 2. Asterisks denote 6 SNPs with a clinal pattern across the whole transect.

Using 10,597 SNPs as input when looking for loci that show departure from neutrality, three different methods including *OutFLANK*, *pcadapt*, and RDA detected 200, 239 and 55 SNPs, respectively. To provide robust inference and avoid reporting false positives we consider only 20 SNPs (PAV) detected by all three methods as systematically linked with local adaptation in *P. sylvestris* (Figure 3A). The frequency of six of those SNPs showed a clinal pattern across the whole range (SNP_15, SNP_148, SNP_1616, SNP_3701, SNP_5039 and SNP_5563), while the rest showed a clinal pattern only within one region, being mostly fixed in the other (Figure 3B,C). Moreover, all of them were found in the RDA to be associated with the RDA1 axis, strongly correlated with mean annual temperature (Figure S8C,D), and allele frequencies at those SNPs showed a linear relationship with this environmental variable, with a mean correlation coefficient R^2^ = 0.56 (range of R^2^: 0.47–0.79), compared to values mean R^2^ = 0.06 for all non‐outlier SNPs (Figure 3C). Results of the GLM analysis with a binomial error provided additional support for this relationship and additionally highlighted the association of individual genotypes with temperature for those SNPs (Figure S9). For all the PAVs we detected strong statistical support for the relationship between genotype and temperature, indicated by extremely low *p* values, and Z‐scores. The majority of PAVs exhibited a strong positive association with temperature, indicating that one homozygote was more likely to occur in warmer environments, compared to the alternative homozygote in this locus (Table S4). For instance results of the GLM for SNP_15 (β estimate = 0.37, odds ratio = 1.45, *p* value = 1.20 × 10^−21^) and SNP_10451 (β estimate = 0.55, odds ratio = 1.74, *p* value = 1.54 × 10^−10^) indicate an increased probability of having one homozygote over the alternative of 45% and 74% for every unit of temperature increase, respectively. It is worth noting that among SNPs selected as outliers by RDA (55), all but two were strongly correlated with mean annual temperature (53 SNPs), and those two SNPs showed the highest correlation with temperature seasonality and precipitation of the wettest quarter. (Figure S8C). Our BLAST analysis provided some insight into the function of PAV; however the majority of them were assigned to unknown or hypothetical proteins. Three SNPs did not produce any significant BLAST hits, further highlighting the challenges of functional annotation in this species. Only a few PAVs showed homology to known protein‐coding genes in other species, including transcriptional regulators, enzymes involved in metabolic pathways, and structural proteins (Table S5).

We also wanted to contrast the relatively weak interregional population structure that likely reflects the background neutral variation with SNPs showing departure from neutrality, to find genetic variants, that may be uniquely favored in those two regions. Using genomic outlier scans (*OutFLANK* and *pcadapt*) we found 66 such shared outlier SNPs in Poland and 104 in Finland (Figure S10A,B), nearly all of which (except four SNPs in Finland) overlapped with SNPs identified for the entire transect using the same methods (Figure S10C). When examining the intersection between outliers detected by genomic scans and those identified using RDA, we found six overlapping SNPs in Finland but none in Poland. This suggests that environmental selection pressures may be stronger or more distinct in Finland, leading to the convergence of signals across different statistical approaches, whereas in Poland, selection signals may be more subtle or confounded by other factors.

The RDA analysis was also used to explain the partitioning of the population genetic variation between the effects of environment and geography. After removing the highly correlated variables, only eight environmental (mean_temp, mean_dr, temp_s, perc_dry_m, perc_wet_q, ph, wet and carbon) and 10 geographic dbMEM were included in the model (Figure S8A,B, Table S2). Together, environment and geography explained 18.8% of the total genetic variance (*R* ^2^ = 0.045 and 0.110, *p* < 0.001; “combined fractions” in Table S6). Partial redundancy analysis showed, that 12% of variance was confounded between the two matrices, and that when constraining one matrix with the other, environment explained much less observed genomic variation than geography (0.1% vs. 6.7%, respectively, “individual fractions” in Table S6). We then performed RDA using outlier SNPs: 151 SNPs detected by genomic outlier scans (*OutFLANK* and *pcadapt*) and 20 PAVs. We found that the contribution of environment and geography jointly explained the majority of variation, especially in the case of PAVs leaving only 11.6% of variation unexplained. Surprisingly, in all of those analyses, the explanatory power of environment alone was smaller, than the exclusive impact of geography (0.1% vs. 6.7% for all SNPs; 3.5% vs. 17.5% for genomic scans; 6.5% vs. 13% for PAVs, Table S6).

### 3.3 Predicting Genomics Offset for Future Climate Change

**FIGURE 4:**
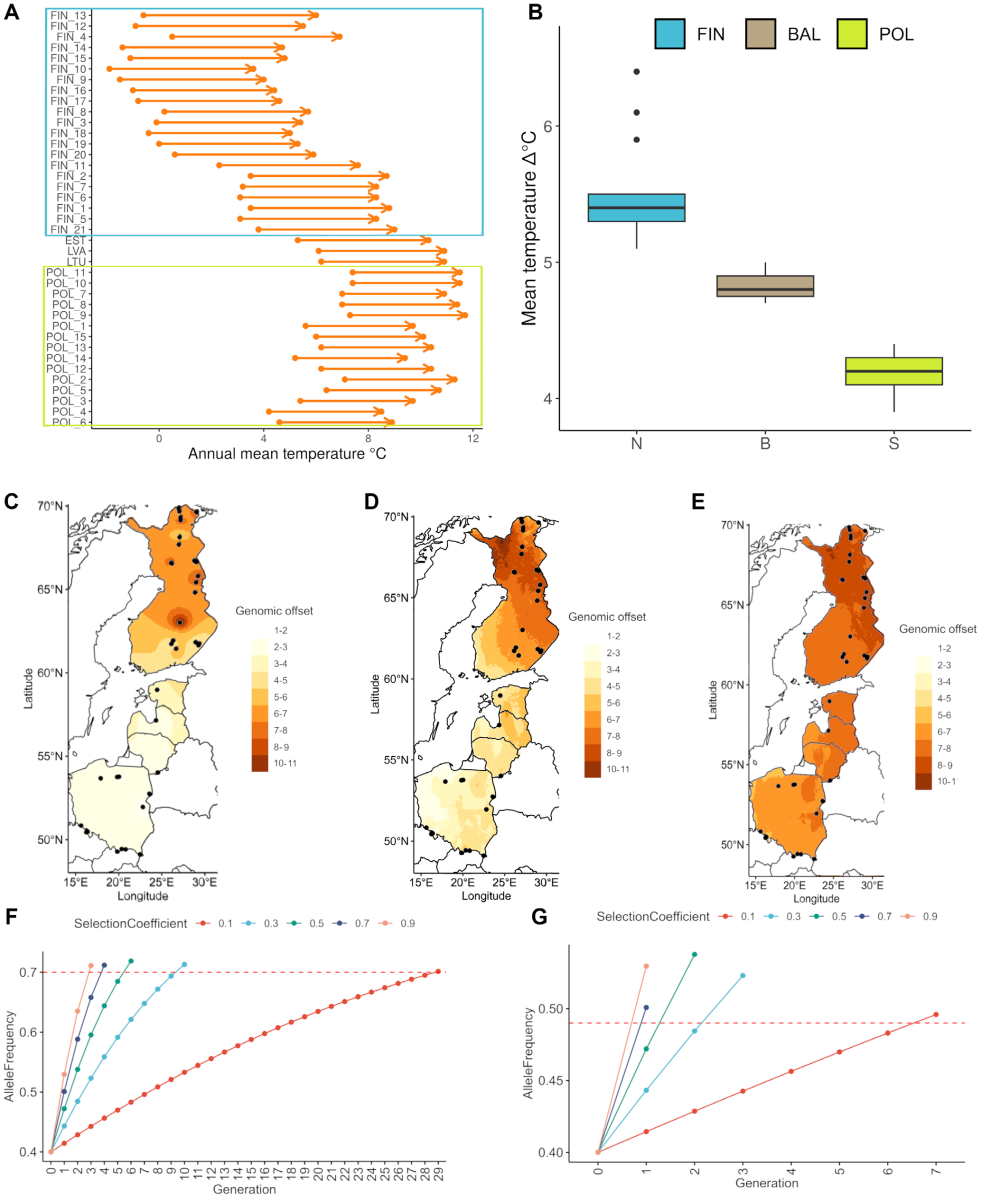
Genomic offset of Scots pine. (A) Predicted change in mean annual temperature in 39 pine populations studied under SSP8.5. The arrow represents ∆°C—the amount of change in mean annual temperature at a given site. (B) Boxplot showing the comparison of mean values of ∆°C between different regions under SSP8.5. (C–E) Spatial projection of genomic offset values across the Scots pine range based on 20 potential adaptive variants (PAVs). Black dots denotes location of studied populations. (C) Projection based on RONA‐RDA method, where mean allele shift across 20 PAV was calculated under the SSP8.5 scenario. See Figure S12 for values under other scenarios. (D) Projection of RDA offset, based on differences in adaptive index for current and future climate under SSP8.5 scenario. (E) Projection of GF (Gradient Forest) offset, displaying predicted multilocus genomic change across environmental gradients under the same scenario. (F, G) Time in generations required for change in allele frequencies assuming only selection of different strength, shown for selection coefficients ranging from 0.1 to 0.9. Both panels show simulation results under SSP8.5 with intermediate allele frequencies (0.4) as starting point. (F) Simulation results for the maximum genomic offset value of ∆*p* = 0.27. (G) Simulation results for the minimum genomic offset value of ∆*p* = 0.06.

Based on the contemporary genotype‐environment association found for SNPs denoted as potentially adaptive, we could predict the amount of genetic change in a population required to keep up with future climate change. The most important factor driving the allele frequency change in our study was mean annual temperature, which is predicted to increase substantially for all studied populations, regardless of the SSP scenario (with the highest increase under the SSP 585 scenario), and the temperature in the north is predicted to increase relatively higher than in the middle latitudes, which is also reflected in our data, and the spatial distribution of genomic offset values (Figure 4A–E). Across methods, the spatial patterns of genomic offset were strikingly similar. RONA‐RDA, RDA, and GF offsets all indicated increasing maladaptation risk towards the northernmost populations, with strong and significant correlations among methods (Figure 4C–E; Figure S11). While the overall geographic trends were consistent, the magnitude of RONA‐RDA offsets was more sensitive to the number of loci included: values shifted when using the expanded SNP panel, reflecting the averaging of allele‐frequency differences across more variants (Figure S12). In contrast, RDA and GF offsets were highly stable across both SNP sets, producing nearly identical patterns (Figure S12).

Predicted allele shifts were observed in all populations across all loci and climate scenarios, with only small increases in magnitude between SSPs, which resulted in a similar trend for genomic offset (SSP 126 < SSP 245 < SSP 585; Figure S11A,C; Table S7). The highest genomic offset values were reported for SSP 585 with RONA‐RDA for FIN11 (0.27) and the lowest for POL4 and POL 12 (0.06), while with RDA offset: FIN 17 (45.07) and POL 7 (33.65), respectively. Generally, the genomic offset increase mirrored the temperature trend (with the Northernmost population having the highest values), with some exceptions in Finland and Poland (Figure 4C,D). The mean genomic offset values for Finland and Poland under SSP 585 were as follows: 0.1824 versus 0.0834 for RONA‐RDA, 42.75 versus 35.21 for RDA offset, and 1.24 versus 0.95 for GF offset.

Because genomic offset projections were highly correlated across the three methods, we used the RONA‐RDA‐based estimates, expressed as the average change in adaptive allele frequencies, to calculate the time required for shifts in allele frequencies under selection alone. The highest genomic offset values reported for SSP 585 with RONA‐RDA were 0.27 (FIN11) and the lowest 0.06 (POL4 and POL 12) and those values were used as the upper and lower bounds of allele frequency change in our simulations. We obtained estimates for the time required for such change under selective pressure ranging from extremely high (*s* = 0.9) to moderately low (*s* = 0.1), and assuming moderate starting allele frequencies (*p* = 0.4). For maximum genomic offset under SSP 585 (the most extreme climate change scenario) our time was bound between 3 and 29 generations (75–725 years) and for the minimum genomic offset value it was 1–7 generations (25–225 years), with slightly lower estimates for the milder climatic change scenario modeled in SSP 126 (Figure 4F,G). Although we assumed for simplicity the same starting allele frequencies for populations in Poland and Finland, this was not often the case—for some PAV, many populations from Poland had frequencies ~0.7–0.8. To account for these differences, we additionally show the relationship between starting allele frequencies, the strength of selection and given (∆*p*). Regardless of the strength of selection, the required time could be substantially longer when starting from low or approaching high allele frequencies (Figure S13).

## 4 Discussion

Our work advances understanding of the mechanisms shaping the distribution of genetic variation in Scots pine, a keystone economically important tree species in Eurasia forests. We show that geography primarily structures the neutral genetic background of populations, whereas adaptive responses are strongly driven by temperature. Variance partitioning of the full SNP set explained only a modest fraction of genome‐wide variation, but the association of putatively adaptive loci with temperature was much stronger. This provided the basis for our genomic offset predictions, which identify populations likely to experience greater allele‐frequency shifts under future climates. In line with the IPCC Sixth Assessment Report (Intergovernmental Panel on Climate Change 2023) and widely used framework for evaluating species' vulnerability to climate change (Foden et al. 2019), we define vulnerability as the propensity to be adversely affected, shaped by sensitivity and adaptive capacity. Our genomic offset estimates capture exposure and sensitivity through predicted allele‐frequency shifts, but do not measure adaptive capacity directly. Although the term genomic vulnerability has sometimes been used synonymously in the literature (Bay 2018; Fitzpatrick and Keller 2015; Rellstab et al. 2021), we deliberately avoid this conflation and instead use genomic offset as a relative indicator of potential maladaptation under climate change, while recognizing that it represents only part of the broader vulnerability framework. This distinction is critical for management: offset highlights populations likely to experience strong genetic shifts, but not their ultimate persistence or resilience. Accordingly, the geographic distribution of adaptive alleles and their predicted frequency changes under SSP scenarios therefore point to the need for active management to safeguard the resilience of Scots pine populations in the face of ongoing climate change.

The analyzed populations were previously included in a study addressing neutral genetic variation and demographic history of Scots pine (Łabiszak and Wachowiak 2023). They span a broad environmental gradient and represent one of the best‐documented examples of adaptive variation in frost tolerance and phenology within this species (Savolainen et al. 2007). Despite environmental differences and distinct demographic histories, our analysis shows that populations share much of their neutral genetic variation, with nearly identical allele‐frequency spectra at most loci. Neutral variation is also distributed relatively uniformly across the range (mean *F*_ST_≈0.017), indicating low genetic differentiation. Our results corroborate other reports of missing populations differentiation in Scots pine—referring to the fact of unusually low genetic differentiation compared to species with similar distribution ranges, which is likely attributed to the combined effects of multiple range shifts experienced in the past, large effective population sizes, and high levels of gene flow (Pyhärjärvi et al. 2020; Wachowiak et al. 2023; Żukowska and Wachowiak 2017; Milesi et al. 2024). Postglacial recolonization also played a significant role in shaping the observed allele frequency gradients in Scots pine along the studied transect, as indicated by the cline‐like pattern in ancestry found by LEA and PCA analysis. Overall, variation could be partitioned between isolation by distance (IBD) and isolation by environment (IBE). These spatial processes are not mutually exclusive and can interact by reducing gene flow among ecologically divergent and geographically distant populations (DeWoody et al. 2015; Papadopulos et al. 2014). In *P. sylvestris* , IBD dominates neutral divergence, while IBE leaves a stronger signal at outlier loci, with only 15% of the variation unexplained. Certain loci under selection, or tightly linked to adaptive regions, may therefore be more resistant to the homogenizing effects of gene flow (Tigano and Friesen 2016), a pattern was also reported in other pine species with comparable ranges and glacial histories (Nadeau et al. 2016; Nadeau et al. 2015; Xia et al. 2018).

Scots pine, like other keystone forest tree species, has been the focus of numerous quantitative and population genetic studies aiming to uncover the genetic basis of adaptive trait variation, including phenology, cold tolerance, responses to drought or waterlogging (Alberto et al. 2013; Savolainen et al. 2007; Kujala 2017; Salmela et al. 2013; Hurme et al. 1997; Donnelly et al. 2018; Wachowiak et al. 2018; Semerci et al. 2017). These traits often show clinal patterns across the range, and common garden experiments confirm both their high heritability and strong signatures of local adaptation (Pyhärjärvi et al. 2020; Giertych and Mátyás 1991). Some quantitative genetic studies suggested the presence of genes with a strong phenotypic effect related among others to cold tolerance and phenology (e.g., reviewed in (Neale and Savolainen 2004)). By contrast, association genetic studies generally indicate that adaptive variation is shaped by many loci of small effect, consistent with polygenic architecture (Hall et al. 2021; Kujala 2017; Tyrmi et al. 2020; Wachowiak et al. 2009; Kujala and Savolainen 2012).

The polygenic nature of many climate‐associated traits often produces subtle allele‐frequency shifts that are difficult to capture with any single outlier‐detection method (Whitlock and Lotterhos 2015). To address this, we combined complementary methods, examining different aspects of the association between genotype and environment. Specifically, we analyzed patterns of differentiation based on *F*_ST_, SNPs underlying population structure, and allele frequencies associated with the multivariate environmental predictors. By focusing on loci consistently identified across all three approaches, we maximized detection power while minimizing false positives, while acknowledging that this conservative strategy may exclude some true adaptive variants. Nevertheless, the consistent patterns observed when applying the broader outlier panel confirm that our conclusions are robust to this trade‐off. These loci contribute meaningfully to patterns of genetic variation across the Scots pine range, as confirmed by our multivariate analyses. In particular, they produced PCA structuring consistent with the full dataset, while neutral SNPs of equal size repeatedly failed to reveal any structure, underscoring that the detected outliers capture biologically relevant adaptive signals.

Growing evidence supports a polygenic basis of local adaptation in non‐model organisms, including forest tree species like poplars, oaks and beeches (Sang et al. 2022; Yuan et al. 2023; Lazić et al. 2024). Climate‐associated traits with a complex genetic architecture shaped in response to environmental gradients through multilocus selection were described in other species including among others humans (Field et al. 2016; Sella and Barton 2019), fruit flies (Barghi et al. 2019) and Arabidopsis (Hancock et al. 2011). In the case of species with a high level of gene flow selection must be high enough to overcome its homogenizing effect to maintain local adaptation (Hancock et al. 2011; Chavarria‐Pizarro et al. 2019). In our study, the variation in all SNPs putatively under selection was highly correlated with mean annual temperature and possibly other environmental variables highly correlated with temperature, but not with precipitation. Temperature serves as a key limiting factor for tree species, especially young individuals, influencing growth patterns and phenology, and ultimately impacting tree performance and survival (Niinemets and Valladares 2006; Salojärvi et al. 2017; Ryan 2010). This suggests that selective pressure from temperature is strong enough to maintain local adaptation in Scots pine despite high gene flow. Assessing the strength of local adaptation is also critical for interpreting genomic offset, as recent work shows that the reliability of offset predictions increases when populations exhibit strong local adaptation. Under such conditions, genomic offset methods perform well, particularly when predicting within environmental conditions similar to those used in model training (Lind and Lotterhos 2025).

The functional interpretation of climate‐associated SNPs in Scots pine remains limited, reflecting both the complexity of conifer genomes and the absence of a high‐quality annotated reference for this species. Given the rapid decay of linkage disequilibrium within the Scots pine genome (LD drops to *R* ^2^ < 0.2 at ~200 bp (Wachowiak et al. 2009; Dvornyk et al. 2002)) and the reduced genome representation method for genotyping used here (SNP array), with only a small fraction of the whole genome likely represented, we can expect a limited number of SNP outliers detected by all three genome‐environment association methods. These rare but concordant variants are strong candidates for causative polymorphisms or loci in tight linkage with them (Neale and Savolainen 2004). Among the annotated variants, several genes point to plausible roles in temperature adaptation. For example, Med15a (SNP_1693) is involved in the transcriptional regulation of stress‐responsive genes, including heat shock proteins, which help plants cope with extreme temperatures (Crawford et al. 2020; Chen et al. 2022; Cooper et al. 2025). MOR1 (SNP_3156) plays a role in the organization of microtubules, and temperature‐sensitive mutants show severe developmental defects at high temperatures, indicating their importance in maintaining cellular integrity under thermal stress (Whittington et al. 2001). Additionally, F3H (SNP_3497) contributes to flavonoid biosynthesis, a pathway known for its protective role against oxidative stress and UV radiation, which can be exacerbated by temperature fluctuations (Liu et al. 2013). The NAC transcription factor JA2L (SNP_7719) has been linked to abiotic stress responses, including temperature variation, through its role in regulating stress‐induced gene expression (Diao et al. 2020). These findings suggest a role in thermal adaptation, but functional validation is required to establish fitness effects. Taken together, these results align with the broader evidence for polygenic adaptation. Even though only a limited number of concordant SNPs were detected, their strong environmental associations, functional plausibility, and consistency across methods strongly suggest that local adaptation in Scots pine is maintained by selection acting on numerous loci of small effect. In addition to putative structural variation in genes underlying local adaptation, variants involved in regulatory mechanisms, gene expression patterns, or epigenetic changes could be equally important (Verhoeven et al. 2016; Sork 2017). Further studies that will take advantage of the availability of the complete genome sequence of Scots pine and methods in functional genomics and plant molecular biology are needed to fully explore those possibilities.

In a rapidly changing environment, forest tree populations face limited options for survival. These options include tracking the shifting climatic niche through migration, relying on pre‐existing genetic variation and selection to adapt to novel conditions in their current location (Aitken et al. 2008). Life history traits, especially pollen and seed dispersal rates, serve as limiting factors for effective migration, and the actual rates are quite low for many tree species (Meier et al. 2012; McLachlan et al. 2007). A significant discrepancy between actual and predicted rates of migration to track future conditions has been reported, raising concerns about the capacity of many tree species to rely on natural migration alone (Dyderski et al. 2018; Zhu et al. 2012; Malcolm et al. 2002). Our genomic offset estimates underscore this challenge. Predicted allele‐frequency shifts required for adaptation under the RONA framework are relatively large, with mean values highest in the northern part of the transect across all SSP scenarios. For comparison, allele‐frequency changes of ~0.01 per generation have been observed at neutral and adaptive loci in other tree species (Dauphin et al. 2021), whereas our predicted shifts are an order of magnitude greater. This indicates that closing the offset gap through natural selection alone will be difficult for Scots pine populations.

Our simulation results reinforce this conclusion by showing that the time required for allele‐frequency shifts in Scots pine ranges from ~25 to more than 700 years, depending on the genomic offset value. The dynamics were notably influenced by the strength of selection, with a higher selection coefficient resulting in quicker shifts, and the initial allele frequency, wherein shifts occurred more rapidly at intermediate frequencies. While there is a possibility that climate change‐induced mismatches could lead to heightened mortality rates (indicative of high selection pressures), a meta‐analysis investigating selection strength within natural populations delivered a mean selection coefficient of *s* = 0.135 (Thurman and Barrett 2016). The scarcity of studies reporting very high selection coefficients (*s* > 0.5) suggests that the time required for allele frequency changes may need to be long (Thurman and Barrett 2016). To reflect this, our modelling approach was deliberately structured as a conservative baseline, focusing solely on the effect of selection. Processes such as gene flow, drift, or demographic buffering can interact with selection, but are unlikely to accelerate adaptation. In Scots pine effective gene flow is strongest among nearby populations (Kujala and Savolainen 2012; Robledo‐Arnuncio and Gil 2005) and for SNPs underlying our offset estimates, this means gene flow is more likely to redistribute standing variation locally than to consistently introduce adaptive alleles from distant environments. Moreover, in the absence of adaptive introgression, gene flow can introduce both maladaptive and beneficial alleles, and its net contribution to adaptation is therefore expected to be variable and generally limited on average (Tigano and Friesen 2016; Lenormand 2002; Kremer et al. 2012). Nevertheless, incorporating these processes would most likely extend, rather than shorten, the estimated timeframe. Thus, it is evident that standing genetic variation in many places may become maladapted to rapidly changing environments, and alternative approaches as compared to natural regeneration might be needed to ensure the resilience of Scots pine in the face of climate change (Aitken et al. 2008; Aitken and Whitlock 2013; Palik et al. 2022).

Overall, our analyses indicate that Finnish populations, especially those located above the Arctic Circle, could be more harmed as a consequence of increased warming of the Arctic region (Rantanen et al. 2022). In contrast, the results of species distribution changes in response to future conditions have generally predicted a substantial northward range shift in the case of Scot pine, and more optimal conditions to grow at higher latitudes (Dyderski et al. 2018). This apparent contradiction may be reconciled by considering the need for turnover: pines, currently growing in affected areas, will likely be replaced by those migrating from lower latitudes. At finer spatial scales, within individual regions, there is a slight variation, and the relationship between latitude and genomic offset is weaker, particularly in Poland. Importantly, these geographic trends were consistent across RONA‐RDA, RDA, and GF, and remained robust when using the expanded SNP set. While RONA‐RDA values showed somewhat greater variance due to averaging across many loci, both RDA and GF converged on nearly identical predictions. This convergence across methods and marker sets is in line with recent empirical and theoretical work demonstrating the reliability of genomic offset as a comparative measure of exposure (Lind and Lotterhos 2025; Fitzpatrick et al. 2021; Gain et al. 2023; Francisco et al. 2025).

Conceptually, however, genomic offset assumes populations are close to adaptive equilibrium with their present climate. Yet evidence from other tree species shows this is not always the case; for example, *Quercus lobata* exhibits an adaptational lag to temperature (Browne et al. 2019), and European forests more broadly show delayed adaptation to current conditions (Fréjaville et al. 2020). In such cases, warming may initially relieve climatic constraints and even increase fitness in some northern populations, despite offset values suggesting substantial allele‐frequency shifts. These short‐term deviations, however, do not diminish the value of offset, which is best understood as identifying populations requiring the steepest relative genomic changes. Our genomic offset estimates therefore highlight strong allele‐frequency shifts expected under climate change, but we emphasize that these values should not be interpreted as direct measures of fitness. Offset quantifies exposure and sensitivity, whereas adaptive capacity, which includes processes such as plasticity, dispersal, and demographic buffering, lies outside its scope. These processes can mitigate or amplify the vulnerability of Scots pine populations, so our results capture only part of the broader picture. Nevertheless, growing evidence indicates that offset provides reliable signals of potential maladaptation. For example, common garden experiments in balsam poplar (Fitzpatrick et al. 2021) and mortality patterns in maritime pine (Archambeau 2024) both aligned with offset predictions, and theoretical studies support its value as a comparative measure of exposure and sensitivity (Gain et al. 2023). We therefore present genomic offset as a robust and informative measure for identifying populations most likely to undergo strong genetic shifts, while acknowledging that empirical validation under future climatic conditions in Scots pine remains a crucial next step.

The presented research provides an assessment of genomic offset across Scots pine populations, highlighting the magnitude of allele‐frequency change required for adaptation. Even under the mild prognosis of SSP 126, these predicted shifts are substantial, suggesting that natural adaptive responses alone will be insufficient without external assistance. These results directly inform forest management by pointing to populations that may struggle to track rapid climate change without intervention. One potential approach is forest assisted migration (FAM), which involves transferring existing genotypes from one place within the species range to an area of interest, where the projected environmental condition matches those already present in the donor place (Nagel et al. 2017; Park and Talbot 2018; Williams and Dumroese 2013; Pedlar et al. 2012). While FAM focuses on intraspecific transfers, assisted migration as a broader concept also includes moving species beyond their historical range to facilitate climate‐driven shifts. However, genomic offset predictions are most informative within the current climatic envelope of the species and may have reduced reliability when extrapolated to areas that could experience novel or extreme environmental conditions in the future, where genotype–environment relationships may no longer hold (Aitken and Bemmels 2016). Thus, before any implementation, empirical validation is essential. Provenance trials and progeny testing are needed to confirm that predicted adaptive genotypes perform as expected in target environments (Capblancq et al. 2020). Careful selection of the potential source population for assisted migration suited for each location and adequate policy adjustments are needed (Palik et al. 2022). In addition, biotic interactions and other factors influencing sensitivity, such as the possible accumulation of deleterious mutations, should not be overlooked. Especially challenging, given the vast range of Scots pines, are the existing regulations regarding the maintenance of provenance regions and the transfer of reforestation material in different countries. Such regions were delimited to maintain unique ecotypes and/or to protect locally adapted populations. As shown in the most recent Scots pine study (Lasek et al. 2024), careful analysis of the potential revision of such regulations is necessary (McKenney et al. 2009; Karasov‐Olson et al. 2021). Given that the predictive power of genomic offset models declines when applied to highly novel conditions, assisted migration strategies should prioritize transfers within the species' ecological tolerances to maximize success (Lind and Lotterhos 2025). In addition, long‐term monitoring and adaptive management strategies are crucial to evaluate the efficacy of FAM interventions. Despite the challenges, delaying action will likely result in substantial ecological and economic losses. Successful applications of assisted migration in other forest tree species, including black ash, red pine, and eastern white pine (Palik et al. 2022), provide encouraging models. These frameworks can be adapted to Scots pine to develop cost‐effective, evidence‐based climate adaptation strategies that could help mitigate those costs (Janowiak et al. 2014; Brandt et al. 2016; Swanston 2016).

## 5 Conclusions

The study provides a deeper understanding of the genomic basis of local adaptation and the magnitude of genomic offset in Scots pine, a keystone forest tree species that is susceptible to climate change due to fitness loss and genetic maladaptation in large geographic areas. Our landscape genomic analysis identified SNPs associated with environmental gradients, particularly mean annual temperature, underscoring temperature as a primary driver of adaptive differentiation. Predicted genomic offsets are substantial, with simulations indicating that natural selection alone would require hundreds of years to close the gap, far exceeding the pace of expected climate change. Importantly, the spatial trends were consistent across RONA‐RDA, RDA, and GF, and remained robust when using different SNP sets, reinforcing confidence in the general conclusions. To address this challenge, management strategies such as forest assisted migration (FAM) offer a proactive option to enhance resilience by relocating genotypes to climatically suitable regions. Yet, successful implementation demands rigorous validation through common garden experiments, progeny testing, careful selection of source populations, and revision of the existing provenance regions, to ensure its effectiveness. By integrating genome–environment associations with offset modelling, our study advances understanding of climate adaptation in long‐lived forest trees and provides a framework for considering genomic insights in the conservation and management of Scots pine across its range.

## Conflicts of Interest

The authors declare no conflicts of interest.

## Supporting information


**Table S1:** Location of the investigated populations of *Pinus sylvestris* .
**Table S2:**. List of 25 environmental variables selected initially for RDA analysis. Bolded are the variables retained after checking multicollinearity.
**Table S3:** Basic summary statistic for *Pinus sylvestris* populations in the studied transect.
**Table S4:**. Results of the Generalized Linear Model (GLM) analysis assessing the association between potentially adaptive variants (PAVs) and temperature. The model was fitted using a binomial regression with a logit link function, where the log odds of genotype occurrence were modeled as a function of temperature. See Figure S9 for visual representation of those results with boxplots.
**Table S5:**. Results of BLAST analysis of 20 PAV in Scots pine. The analysis was conducted using transcriptomic regions containing the focal single nucleotide polymorphisms (SNPs) as queries. For each SNP, the corresponding Axiom ID, transcriptomic region, BLAST accession number, and identified gene product are reported.
**Table S6:**. Redundancy analysis (RDA) to partition among population genetic variation (F) in *Pinus sylvestris* into environment (env.), geography (geog.) and their combined effects, shown in the table as measured by adjusted R^2^. The proportions of the variation that were exclusively attributed to environment or geography are highlighted in light grey. The individual fractions of the variation that were confounded between various combinations of these two components are highlighted in dark grey.
**Table S7:**. Genomic offset values for Scots pine populations under three climate change scenarios (SSP 126, SSP 245, and SSP 585), estimated using three approaches: (i) RONA‐RDA, which quantifies predicted offset as the mean allele‐frequency shift required under future climates; (ii) RDA offset, based on redundancy analysis; and (iii) Gradient Forest offset, which calculates the multivariate environmental distance between present and future climates weighted by the importance of allele–environment associations inferred from regression trees. Populations are ordered by latitude from north to south.
**Figure S1:** (A) European part of Scots pine distribution range, with sampled populations across environmental transect from Finland to Poland. Individual populations are numbered consistently with Tables S1 and S2. Scots pine range is depicted in green. (B) Ordination of 39 Scots pine populations in environmental space. Light grey dots: global Scots pine distribution (distribution based on EU forest database and associated climate data based on Wordclim). Dark grey dots: distribution of Scots pine in the whole studied transect. The populations used in this study are denoted as bigger points, colour‐coded according to Figure 2. Population numbers match those in Table S1.
**Figure S2:** Heatmap of pairwise F_ST_ values between analysed populations of Scots pine calculated using whole SNP set.
**Figure S3:** Results of the IBD and IBE analyses. (A) The Mantel test scatterplot of IBD shows a linearized measure of genetic distance (FST/(1‐FST)) as a function of geographic distance (B). The Mantel test scatterplot of IBE shows linearized measure of genetic distance (FST/(1‐FST)) as a function of environmental distance based on eight non‐correlated variables.
**Figure S4:** Cross entropy between 10 different runs for each K in LEA plotted vs. number of ancestral populations. The optimal number of clusters is detected by the first significant drop of cross entropy at K = 4, and the second drop is noted at K = 10.
**Figure S5:** The proportion of ancestry of each individual at 39 sites inferred using LEA for K = 2–10. Different colours of the bars correspond to the inferred ancestry. The sites are ordered from north to south.
**Figure S7:** Comparison of PCA based on different numbers of SNPs included. (A) PCA based on all 440 samples and all SNPs after LD pruning (6995 SNPs—identical to the PCA in Figure 2C). (B) PCA based on all 440 samples and putatively neutral SNPs (6975 SNPs, LD pruning, PAVs excluded). (C) PCA based only on PAVs (20 SNP, LD pruning) (D) Correlation between PC scores from PCA with all SNPs (6995) and with PAV SNPs only (20) with 1:1 identity line. Due to the PC scores being centered, this method can estimate how similar are two different PCA runs. The correlation was rather weak, but statistically significant, with R^2^ = 0.221. We used permutation test based on random resampling of 20 SNPs from the whole dataset 10,000 times and calculated correlation for each resampling. The *p* value based on the permutation test was 0.0274, since randomly selected SNPs do not explain observed population structure.
**Figure S8:** (A) Correlations between the environmental variables selected for redundancy analysis. (B) The results of RDA analysis on the ordination plot of the first two RDA axes. The points represent the populations colour coded as in Figure 2, while the blue arrows represent the environmental predictors. The relative arrangement of the points and arrows on the plot represents their relationship to the RDA ordination axes. (C) Histograms of 55 SNPs detected by RDA and their correlations with the associated environmental variables. The vertical black line denotes a cutoff point of low correlation strength (none of the SNPs were found to be below that threshold); Abbreviations—meantemp: annual mean temperature; meandr: mean diurnal range; temps: temperature seasonality; percdrym: precipitation of driest month; percwetq: precipitation of wettest quarter; ph: top soil pH, wet: number of days receiving ≥ 0.1 mm precipitation; carbon: Organic carbon content. (D) Screeplot showing the variance explained by successive RDA axes. The first axis captured the majority of the constrained variance and was therefore the main focus for outlier detection.
**Figure S9:** Correlation between genotype and mean annual temperature using a generalized linear model (GLM) with a binomial error for PAV SNPs. On the x‐axis genotypes are coded as 0,1,2—for homozygote, heterozygote and alternative homozygote; while on y‐axis, temperature is in Celsius.
**Figure S10:** Venn diagram with the number of SNPs detected as outlier using only genomic outliers scans, separately in both regions. (A) Outliers SNPs detected in Finland. (B) Outliers SNPs detected in Poland. (C) Venn diagram with the number of SNPs concordant between two genomic outliers scans in comparisons between Poland (POL), Finland (FIN) and across whole studied transect (ALL).
**Figure S11:** (A–C) Genomic offset under three future climate scenarios (SSP126, SSP245, SSP585) based on 20 potential adaptive variants (PAVs) identified across GEA methods. (A) The results of the genomic offset based on RONA‐RDA methods. Genomic offset score is reflecting the mean amount of allelic shift based on all 20 PAVs. (B) Genomic offset projections based on RDA offset method, reflecting differences in adaptive index for current and future climate under three emissions scenarios. (C) Genomic offset projections based on gradient forest method, calculated as the multivariate environmental distance between present and future climates, weighted by the importance of allele–environment associations inferred from regression trees. (D) Correlations of genomic offset scores from RONA‐RDA and RDA offset methods for three future climatic scenarios. (E) Correlations of genomic offset scores from RONA‐RDA and GF offset methods for three future climatic scenarios.
**Figure S12:** Genomic offset estimates under three climate scenarios (RCP2.6, RCP4.5, RCP8.5) using different methods and SNP panels. (A–C) Spatial projections of genomic offset calculated from the broader outlier panel (164 SNPs detected by at least two independent methods). Methods: (A) RONA‐RDA, (B) RDA offset, (C) Gradient Forest. (D–F) Correlations between genomic offset values derived from the broader outlier panel (164 SNPs) and the conservative panel of 20 PAVs. Methods: (D) RONA‐RDA, (E) RDA offset, (F) Gradient Forest.
**Figure S13:** Number of generations required for the shift in allele frequency under selection starting from different initial allele frequencies. The frequency change equal to the minimum value (A) and maximum value (B) of genomic offset (0.06 and 0.27, respectively) was simulated under different selection coefficients (s in range 0.1–0.9) starting from different initial frequencies (0.01–0.9) with an increment of 0.01. Left: boxplot of the mean number of generations required for the shift at different values of s. Right—relationship between starting allele frequency and number of generations to shift allele frequency under different selection coefficients.

## Data Availability

The datasets generated and analysed during the current study are available at: https://doi.org/10.5061/dryad.05qfttfdd.
