# Supplementary material for: Adaptive Potential and Genomic Vulnerability of Keystone Forest Tree Species to Climate Change: A Case Study in Scots Pine
Source: Evol Appl. 2025 Dec 5;18(12):e70180. doi: 10.1111/eva.70180 (PMC12680428; doi:10.1111/eva.70180)

Supplementary Materials

Adaptive potential and genomic vulnerability of keystone forest tree species to climate change: a case study in Scots pine

Table S1 Location of the investigated populations of *Pinus sylvestris*.

Acr.	Pop #	Location	N	Lat.	Long.	Acr.	Pop #	Location	N	Lat.	Long.
**POL1**	1	Poland, Chojnik	10	50.83	15.63	**FIN3**	21	Finland, nr Rovaniemi	109	66.57	26.21
**POL2**	2	Poland, Szczeliniec	23	50.43	16.23	**FIN4**	22	Finland, nr Kielajoki	14	69.65	29.07
**POL3**	3	Poland, Pusta Wielka	9	49.40	20.82	**FIN5**	23	Finland, Utraslahti	10	61.66	29.30
**POL4**	4	Poland, Koryciska Wielkie	10	49.27	19.83	**FIN6**	24	Finland, Punkaharju	10	61.76	29.39
**POL5**	5	Poland, Pieniński National Park	11	49.42	20.36	**FIN7**	25	Finland, Savonlinna	10	61.84	28.96
**POL6**	6	Poland, Tarnawa	9	49.10	22.49	**FIN8**	26	Finland, Rovaniemi ACHA	10	66.60	26.12
**POL7**	7	Poland, Woziwoda	10	53.67	17.91	**FIN9**	27	Finland, Ivalontie	10	67.71	27.02
**POL8**	8	Poland, Hajnówka	8	52.74	23.58	**FIN10**	28	Finland, nr Palonoja	10	68.15	27.10
**POL9**	9	Poland, Liski reserve	11	51.95	22.82	**FIN11**	29	Finland, nr Ahmaniemi	10	63.03	27.15
**POL10**	10	Poland, Miłomłyn	5	53.75	19.83	**FIN12**	30	Finland, Utsjoki	10	69.70	27.08
**POL11**	11	Poland, Tabórz reserve	9	53.77	20.04	**FIN13**	31	Finland. Usjoentie	10	69.88	27.00
**POL12**	12	Poland, Niknąca Łąka	10	50.47	16.39	**FIN14**	32	Finland, Kamaen	10	69.34	27.22
**POL13**	13	Poland, Głowa Króla	10	50.48	16.38	**FIN15**	33	Finland, nr Tuuruniemi	9	69.17	27.22
**POL14**	14	Poland, Skalniak	11	50.47	16.30	**FIN16**	34	Finland, Salla Reindeer Park	9	66.74	28.82
**POL15**	15	Poland, Błędne Skały	12	50.48	16.29	**FIN17**	35	Finland, nr Kaarti	11	66.66	29.06
**LTU**	16	Lithuania, Čepkeliai	20	54.02	24.55	**FIN18**	36	Finland, nr Raatepuro	10	65.81	29.24
**LVA**	17	Latvia, Dunezers Lake	10	57.15	24.35	**FIN19**	37	Finland, nr Ahola	10	65.44	29.03
**EST**	18	Estonia, Vardi	10	58.98	24.48	**FIN20**	38	Finland nr Hietakylä	10	64.83	28.90
**FIN1**	19	Finland, Joutsa	20	61.74	26.14	**FIN21**	39	Finland, Kuopio-Helsinki	10	61.45	26.69
**FIN2**	20	Finland, nr Temmes	20	64.69	25.71-						

-Acr. – acronym for the population; Pop # - ordering number N – number of samples analysed, Lat. – latitude (N), Long. – longitude (E)

Table S2. List of 25 environmental variables selected initially for RDA analysis. Bolded are the variables retained after checking multicollinearity.

Variable	Abbreviation	Source	Mean (range)*
**Annual mean temperature (°C)**	**mean_temp**	Worldclim[1]	3.20 (-1.9–7.4)
**Mean diurnal range (°C, Mean of monthly (max temp – min)**	**mean_dr**	Worldclim[1]	8.03 (6.3-9.5)
Isothermality (Dimensionless, ×100)	isoterm	Worldclim[1]	2.46 (2.1-3)
**Temperature seasonality (standard deviation ×100) (°C)**	**temp_s**	Worldclim[1]	837.93 (676.2-996.3)
Max temperature of warmest month (°C)	max_t_warm	Worldclim[1]	19.95 (14.6-24)
Min temperature of coldest month (°C)	min_t_cold	Worldclim[1]	-12.19 (-20- -6.6)
Temperature annual range (°C)	temp_ar	Worldclim[1]	32.15 (26.6-38.3)
Mean temperature of wettest quarter (°C)	mean_t_wet	Worldclim[1]	13.52 (9.2-17.7)
Mean temperature of driest quarter (°C)	mean_t_dry	Worldclim[1]	-4.41 (-9.5-2)
Mean temperature of warmest quarter (°C)	mean_t_warm	Worldclim[1]	15.32 (8.8- 19.65)
Mean temperature of coldest quarter (°C)	mean_t_cold	Worldclim[1]	-7.64 (-14.6- -2.5)
Annual precipitation (mm)	perc	Worldclim[1]	623.38 (407-1134)
Precipitation of wettest month (mm)	perc_wet_m	Worldclim[1]	84.51 (64-172)
**Precipitation of driest month (mm)**	**perc_dry_m**	Worldclim[1]	30.13 (17-54)
Precipitation seasonality (coefficient of variation) (mm)	perc_s	Worldclim[1]	34.79 (28-46)
**Precipitation of wettest quarter (mm)**	**perc_wet_q**	Worldclim[1]	234.08 (165-457)
Precipitation of driest quarter (mm)	perc_dry_q	Worldclim[1]	96.46 (55-170)
Precipitation of warmest quarter (mm)	perc_warm	Worldclim[1]	230.54 (165-457)
Precipitation of coldest quarter (mm)	perc_cold	Worldclim[1]	110.28 (63-180)
Growing degree days heat sum above 5°C (°C-days)	gdd	Climatrends R package[2]	1501.48 (692.3-
**Top soil pH (pH units)**	**ph**	Soil ph in Europe[3]	4.85 (3.3-6.2)
**Organic carbon content (g kg^{-1})**	**carbon**	LUCAS 2018 TOPSOIL data[4]	65.64 (18.17-93.25)
Annual mean UV-B (J/m²/day)	ubv	A global UV-B radiation dataset for macroecological studies[5]	973.96 (691.50-
**Number of days receiving ≥0.1 mm precipitation (days)**	**wet**	CR UTS v. 4.07[6]	20.70 (18.1-24.09)
Number of frost days (days)	frs	CR UTS v. 4.07[6]	28.66 (25.03-31)

[1] Worldclim - Fick, S.E. and R.J. Hijmans, 2017. Worldclim 2: New 1-km spatial resolution climate surfaces for global land areas. International Journal of Climatology;

[2] Climatrends R package - Kauê de Sousa, Jacob van Etten and Svein Ø. Solberg (2020). climatrends: Climate Variability Indices for Ecological Modelling. R package version 0.5. https://CRAN.R-project.org/package=climatrends

[3] Soil ph in Europe - 'Map of Soil pH in Europe', Land Resources Management Unit, Institute for Environment & Sustainability, European Commission"2010

[4] LUCAS 2018 TOPSOIL data - Orgiazzi, A., Ballabio, C., Panagos, P., Jones, A., Fernández-Ugalde, O. 2018. LUCAS Soil, the largest expandable soil dataset for Europe: A review. European Journal of Soil Science, 69(1): 140-153. https://doi.org/10.1111/ejss.12499

[5] A global UV-B radiation dataset for macroecological studies - Beckmann M., Václavík T., Manceur A.M., Šprtová L., von Wehrden H., Welk E., Cord A.F. (2014) glUV: A global UV-B radiation dataset for macroecological studies, Methods in Ecology and Evolution, 5: 372–383. doi: 10.1111/2041-210X.12168

[6] CRUTS v. 4.07 - Harris, I., Osborn, T.J., Jones, P. et al. Version 4 of the CRU TS monthly high-resolution gridded multivariate climate dataset. Sci Data 7, 109 (2020). https://doi.org/10.1038/s41597-020-0453-3

* Mean of environmental variables across the studied populations

Table S3 Basic summary statistic for *Pinus sylvestris* populations in the studied transect.

Acr.	Pop #	region	He	Ho	F_{IS}	Acr.	Pop #	region	He	Ho	F_{IS}
POL1	1	POL	0.3365	0.3361	-0.0062	FIN3	21	FIN	0.3126	0.3143	-0.0087
POL2	2	POL	0.3316	0.3292	-0.0059	FIN4	22	FIN	0.3126	0.3219	-0.0260
POL3	3	POL	0.3332	0.3314	-0.0102	FIN5	23	FIN	0.3130	0.3140	-0.0079
POL4	4	POL	0.3319	0.3263	-0.0200	FIN6	24	FIN	0.3137	0.3136	-0.0058
POL5	5	POL	0.3413	0.3337	-0.0227	FIN7	25	FIN	0.3107	0.3122	-0.0097
POL6	6	POL	0.3341	0.3253	-0.0285	FIN8	26	FIN	0.3131	0.3158	-0.0114
POL7	7	POL	0.3427	0.3366	-0.0192	FIN9	27	FIN	0.3124	0.3148	-0.0111
POL8	8	POL	0.3360	0.3300	-0.0222	FIN10	28	FIN	0.3116	0.3127	-0.0084
POL9	9	POL	0.3371	0.3358	-0.0079	FIN11	29	FIN	0.3077	0.3110	-0.0130
POL10	10	POL	0.3452	0.3348	-0.0432	FIN12	30	FIN	0.3112	0.3141	-0.0125
POL11	11	POL	0.3320	0.3294	-0.0112	FIN13	31	FIN	0.3051	0.3132	-0.0235
POL12	12	POL	0.3384	0.3328	-0.0171	FIN14	32	FIN	0.3115	0.3152	-0.0145
POL13	13	POL	0.3383	0.3335	-0.0169	FIN15	33	FIN	0.3099	0.3136	-0.0158
POL14	14	POL	0.3333	0.3261	-0.0221	FIN16	34	FIN	0.3157	0.3156	-0.0077
POL15	15	POL	0.3330	0.3296	-0.0127	FIN17	35	FIN	0.3131	0.3185	-0.0191
LTU	16	BAL	0.3358	0.3333	-0.0072	FIN18	36	FIN	0.3074	0.3139	-0.0224
LVA	17	BAL	0.3404	0.3359	-0.0145	FIN19	37	FIN	0.3127	0.3155	-0.0129
EST	18	BAL	0.3416	0.3344	-0.0223	FIN20	38	FIN	0.3090	0.3174	-0.0264
FIN1	19	FIN	0.3348	0.3339	-0.0036	FIN21	39	FIN	0.3066	0.3117	-0.0185
FIN2	20	FIN	0.3318	0.3335	0.00264						

Acr. – acronym for the population; Pop # - ordering number; He – Expected heterozygosity, Ho – Observed heterozygosity; F_{IS} Fixation index

Table S4. Results of the Generalized Linear Model (GLM) analysis assessing the association between potentially adaptive variants (PAVs) and temperature. The model was fitted using a binomial regression with a logit link function, where the log odds of genotype occurrence were modeled as a function of temperature. See Fig S9 for visual representation of those results with boxplots.

SNP name	β1 Estimate	Odds ratio	Z value	P value
SNP_15	0.37	1.45	9.56	1.20E-21
SNP_148	-0.34	0.71	-9.04	1.63E-19
SNP_1616	0.24	1.28	7.10	1.24E-12
SNP_1693	-0.51	0.60	-7.05	1.78E-12
SNP_3156	-0.24	0.79	-6.17	6.91E-10
SNP_3497	-0.31	0.74	-7.70	1.40E-14
SNP_3701	-0.26	0.77	-6.80	1.04E-11
SNP_5039	-0.18	0.84	-5.46	4.64E-08
SNP_5563	0.24	1.27	6.91	4.96E-12
SNP_7184	-0.57	0.56	-6.07	1.25E-09
SNP_7375	-0.54	0.58	-6.76	1.35E-11
SNP_7447	-0.60	0.55	-6.54	6.24E-11
SNP_7719	-0.70	0.50	-7.87	3.58E-15
SNP_8108	-0.63	0.53	-6.64	3.07E-11
SNP_10333	-0.58	0.56	-6.96	3.43E-12
SNP_10339	-0.63	0.53	-7.17	7.63E-13
SNP_10451	0.55	1.74	6.40	1.54E-10
SNP_10466	-0.59	0.56	-7.02	2.18E-12
SNP_10749	-0.64	0.53	-6.77	1.31E-11

Table S5. Results of BLAST analysis of 20 PAV in Scots pine. The analysis was conducted using transcriptomic regions containing the focal single nucleotide polymorphisms (SNPs) as queries. For each SNP, the corresponding Axiom ID, transcriptomic region, BLAST accession number, and identified gene product are reported.

SNP name	Axiom ID	Transcriptom region*	BLAST acession number	Name of product
SNP_1693	AX-117401102	comp51381_c0_seq1_2511	XM_057996914.2	mediator of RNA polymerase II transcription subunit 15a
SNP_3156	AX-117406274	comp46844_c0_seq1_7025	XM_057991042.2	Cryptomeria japonica protein MOR1 (LOC131056779),
SNP_3497	AX-117407434	comp54394_c0_seq2_221	AY832611.1	flavanone 3-hydroxylase (F3H) mRNA
SNP_3701	AX-117408161	comp42558_c0_seq1_515	XM_057979156.2	magnesium-protoporphyrin IX monomethyl ester [oxidative] cyclase, chloroplastic
SNP_5563	AX-117413987	comp40826_c0_seq1_2679	XM_057970981.2	CBS domain-containing protein
SNP_7719	AX-117426266	comp55619_c0_seq1_1893	XM_058014006.2	NAC domain-containing protein JA2L
SNP_15	AX-117395476	Kevin_555_1_960	no hit	-
SNP_148	AX-117396057	CL1489_1_1_635	ATP68370.1	hypothetical protein
SNP_1616	AX-117400851	comp53920_c0_seq1_2107	BT116427.1	hypothetical protein
SNP_5039	AX-117412400	comp87385_c0_seq1_127	BT124148.1	unknown mRNA
SNP_7184	AX-117420590	comp41931_c0_seq1_303	BT110267.1	unknown mRNA
SNP_7375	AX-117422588	comp41931_c0_seq1_996	BT110267.1	unknown mRNA
SNP_7447	AX-117423398	comp52836_c0_seq1_2023	no hit	-
SNP_8108	AX-117430881	comp54135_c0_seq3_1422	BT113286.1	unknown mRNA
SNP_10333	AX-117442607	comp41931_c0_seq1_1204	BT110267.1	unknown mRNA
SNP_10339	AX-117442657	comp37072_c0_seq1_279	BT110466.1	unknown mRNA
SNP_10451	AX-117443709	comp52150_c0_seq1_379	no hit	-
SNP_10466	AX-117443831	comp54135_c0_seq3_2036	BT113286.1	unknown mRNA
SNP_10479	AX-117444035	comp41931_c0_seq1_848	BT110267.1	unknown mRNA
SNP_10570	AX-117444956	comp37072_c0_seq1_194	BT110466.1	unknown mRNA

*Source of transcriptome data: https://doi.org/10.5285/b6900166-ded6-4f7a-8734-484b6f77b2f1

Table S6. Redundancy analysis (RDA) to partition among population genetic variation (F) in *Pinus sylvestris* into environment (env.), geography (geog.) and their combined effects, shown in the table as measured by adjusted R^2. The proportions of the variation that were exclusively attributed to environment or geography are highlighted in light grey. The individual fractions of the variation that were confounded between various combinations of these two components are highlighted in dark grey.

	All (10597) SNPs		OUTFLANK and pcadapt outlier (151) SNPs[b]		PAV (20) SNPs[c]	
Combined fractions[a]	R^2	*p* (>F)[d]	R^2	*p* (>F)[c]	R^2	*p* (>F)[c]
F~env.	0.045	0.001 ***	0.175	0.001 ***	0.490	0.001 ***
F~geog.	0.110	0.001 ***	0.333	0.001 ***	0.626	0.001 ***
Individual fractions[a]						
F~env. | geog.	0.001	0.467[ns]	0.035	0.309	0.065	0.037 *
F~geog. | env.	0.067	0.002 **	0.175	0.003 **	0.130	0.003 **
F~env.. + geog.	0.120	0.001 ***	0.370	0.001 ***	0.689	0.001 ***
**Total explained[e]**	**0.188**		**0.580**		**0.884**	
Total confounded[e]	0.120		0.370		0.689	
**Total unexplained**	**0.812**		**0.420**		**0.116**	
**Total**	**1.000**		**1.000**		**1.000**	

[a]F = Independent matrix of population alleles frequencies; RDA tests are of the form: F~dependent matrices | covariate matrices. env. = environment (eight climatic variables); geog. = geography (10 dbMem variables).[b]Subsets of SNPs detected by two genetic outlier tests (OUTFLANK and pcadapt. [c] Subset of SNPs detected by all three outlier methods.The number of SNPs for each subset is given in parentheses.
[d]; * = $p < 0.05$; ** = $p < 0.01$; *** = $p < 0.001$. Significance of confounded fractions between climate, geography, or north-south ancestry (dark grey rows) was not tested.
[e]Total explained = total adjusted R^2 of individual fractions (light grey + dark grey rows). Total confounded = Total of individual fractions confounded between climate, geography (dark grey rows).

Table S7. Genomic offset values for Scots pine populations under three climate change scenarios (SSP 126, SSP 245, and SSP 585), estimated using three approaches: (i) RONA-RDA, which quantifies predicted offset as the mean allele-frequency shift required under future climates; (ii) RDA offset, based on redundancy analysis; and (iii) Gradient Forest offset, which calculates the multivariate environmental distance between present and future climates weighted by the importance of allele–environment associations inferred from regression trees. Populations are ordered by latitude from north to south.

POP	RONA-RDA genomic offset			RDA_offset			GF offset		
	RCP26	RCP45	RCP85	RCP26	RCP45	RCP85	RCP26	RCP45	RCP85
FIN13	0.08	0.13	0.17	40.65	40.99	42.03	1.23	1.23	1.21
FIN12	0.17	0.16	0.18	42.03	42.46	43.62	1.31	1.30	1.28
FIN4	0.12	0.18	0.23	37.34	37.62	38.81	1.01	1.07	1.08
FIN14	0.13	0.14	0.16	40.95	41.43	42.77	1.36	1.34	1.31
FIN15	0.11	0.19	0.23	40.71	41.22	42.72	1.30	1.29	1.27
FIN10	0.15	0.14	0.14	41.35	41.92	43.45	1.20	1.23	1.22
FIN9	0.18	0.19	0.20	42.91	43.39	45.01	1.34	1.37	1.36
FIN16	0.17	0.18	0.19	41.60	42.25	43.77	1.17	1.23	1.25
FIN17	0.18	0.19	0.20	42.89	43.59	45.07	1.20	1.27	1.29
FIN8	0.22	0.22	0.22	42.13	42.73	44.25	1.28	1.31	1.31
FIN3	0.18	0.19	0.19	42.35	42.94	44.51	1.30	1.32	1.31
FIN18	0.20	0.21	0.21	41.84	42.63	44.04	1.28	1.31	1.31
FIN19	0.21	0.21	0.21	41.84	42.50	43.79	1.31	1.33	1.33
FIN20	0.19	0.19	0.18	41.81	42.44	43.66	1.12	1.18	1.19
FIN11	0.21	0.25	0.27	40.18	40.68	41.89	1.17	1.22	1.21
FIN2	0.17	0.16	0.16	39.27	39.73	40.87	1.08	1.13	1.14
FIN7	0.18	0.16	0.15	40.16	40.73	41.61	1.17	1.18	1.17
FIN6	0.17	0.15	0.14	41.02	41.63	42.63	1.18	1.20	1.20
FIN1	0.13	0.12	0.12	39.06	39.53	40.62	1.09	1.14	1.15
FIN5	0.13	0.12	0.12	40.71	41.32	42.17	1.18	1.20	1.19
FIN21	0.17	0.16	0.16	39.00	39.60	40.62	1.13	1.17	1.18
EST	0.11	0.11	0.11	35.34	36.01	36.64	0.95	1.02	1.06
LVA	0.08	0.07	0.07	36.04	36.84	37.15	1.00	1.05	1.06
LTU	0.09	0.09	0.09	36.84	37.41	37.46	1.15	1.16	1.13
POL11	0.09	0.09	0.09	34.80	35.24	34.97	1.00	1.03	1.02
POL10	0.10	0.10	0.10	35.03	35.41	35.13	1.00	1.02	1.03
POL7	0.10	0.09	0.09	33.60	33.77	33.65	0.97	0.98	0.97
POL8	0.09	0.07	0.08	35.53	36.06	35.78	1.00	1.06	1.07
POL9	0.07	0.06	0.06	36.78	37.14	36.73	0.94	1.01	1.03
POL1	0.08	0.08	0.09	34.15	33.84	33.74	0.81	0.87	0.88
POL13	0.11	0.11	0.10	34.35	34.09	33.97	0.87	0.90	0.90
POL15	0.08	0.08	0.09	34.67	34.49	34.27	0.91	0.94	0.93
POL12	0.06	0.06	0.06	34.35	34.09	33.97	0.87	0.90	0.90
POL14	0.07	0.07	0.07	34.27	34.14	33.91	0.91	0.94	0.93
POL2	0.06	0.06	0.07	34.85	34.64	34.52	0.94	0.96	0.96
POL5	0.11	0.11	0.11	38.53	38.90	37.86	0.88	0.95	0.97
POL3	0.09	0.09	0.09	37.75	37.95	37.01	0.83	0.90	0.91
POL4	0.06	0.06	0.06	37.62	38.01	37.06	0.72	0.82	0.82
POL6	0.08	0.08	0.08	36.27	36.55	35.51	0.82	0.90	0.91

Figure S1. (A) European part of Scots pine distribution range, with sampled populations across environmental transect from Finland to Poland. Individual populations are numbered consistently with Table S1 and S2. Scots pine range is depicted in green. (B) Ordination of 39 Scots pine populations in environmental space. Light grey dots: global Scots pine distribution (distribution based on EU forest database and associated climate data based on Wordclim). Dark grey dots: distribution of Scots pine in the whole studied transect. The populations used in this study are denoted as bigger points, colour-coded according to Fig. 2. Population numbers match those in Table S1.

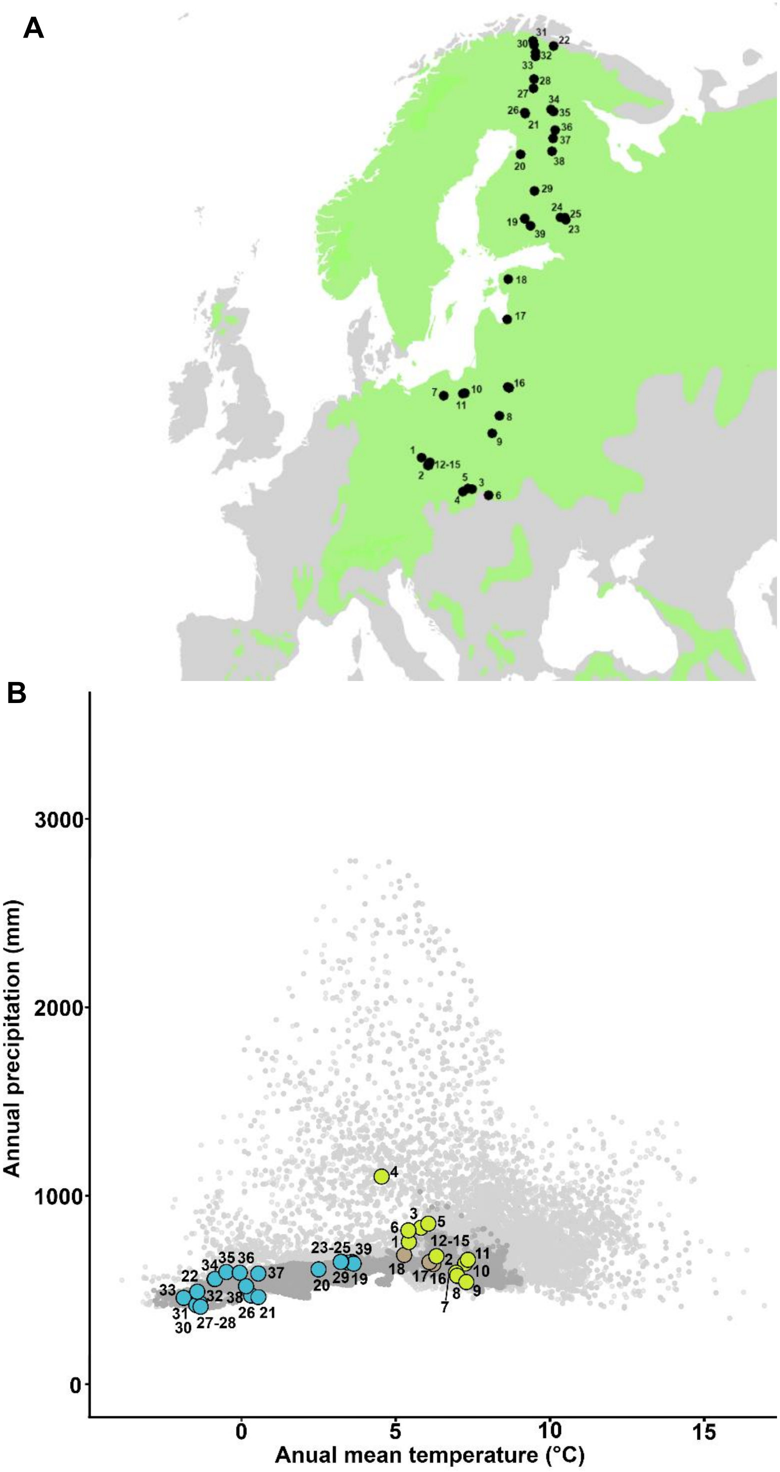

Figure S2. Heatmap of pairwise F_{ST} values between analysed populations of Scots pine calculated using whole SNP set.

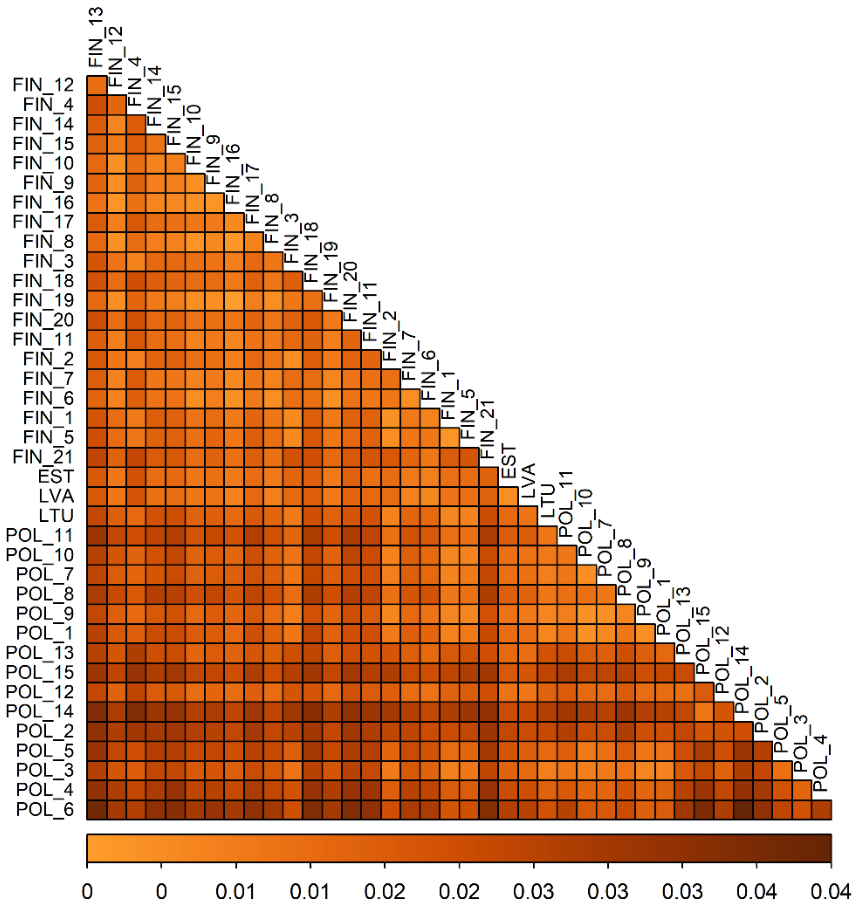

Figure S3. Results of the IBD and IBE analyses. (A) The Mantel test scatterplot of IBD shows a linearized measure of genetic distance (F_{ST} / (1 - F_{ST})) as a function of geographic distance (B). The Mantel test scatterplot of IBE shows linearized measure of genetic distance (F_{ST} / (1 - F_{ST})) as a function of environmental distance based on eight non-correlated variables.

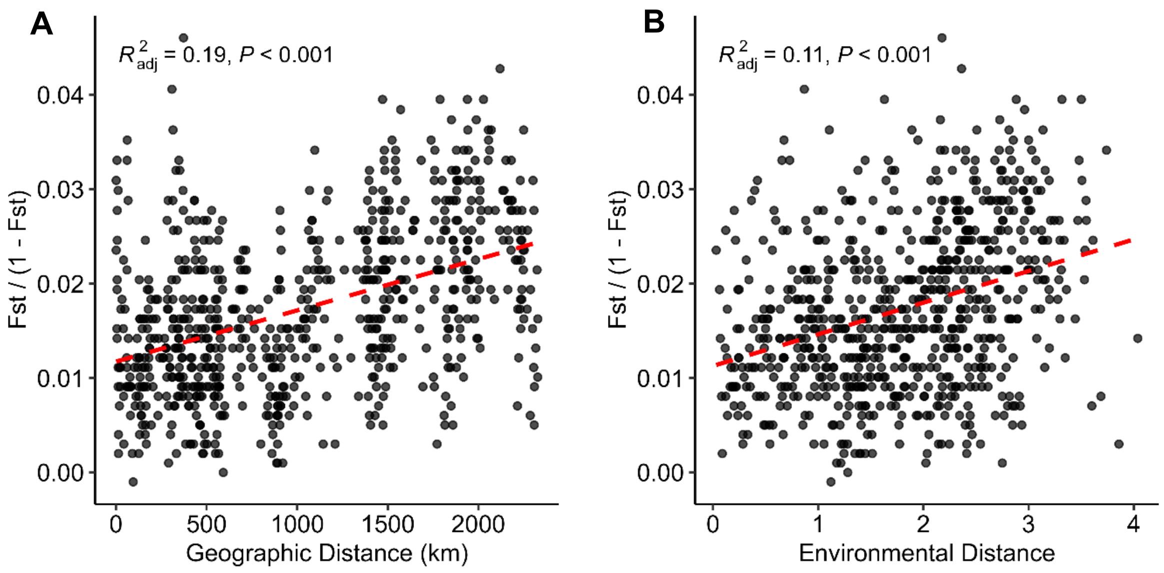

Figure S4. Cross entropy between ten different runs for each K in LEA plotted vs. number of ancestral populations. The optimal number of clusters is detected by the first significant drop of cross entropy at K = 4, and the second drop is noted at K = 10.

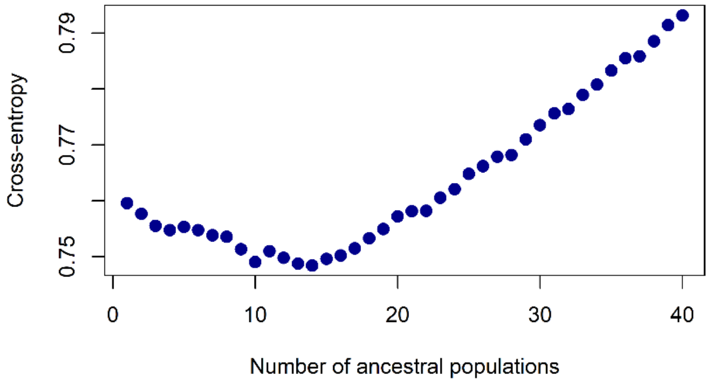

Figure S5. The proportion of ancestry of each individual at 39 sites inferred using LEA for K 2-10. Different colours of the bars correspond to the inferred ancestry. The sites are ordered from north to south.

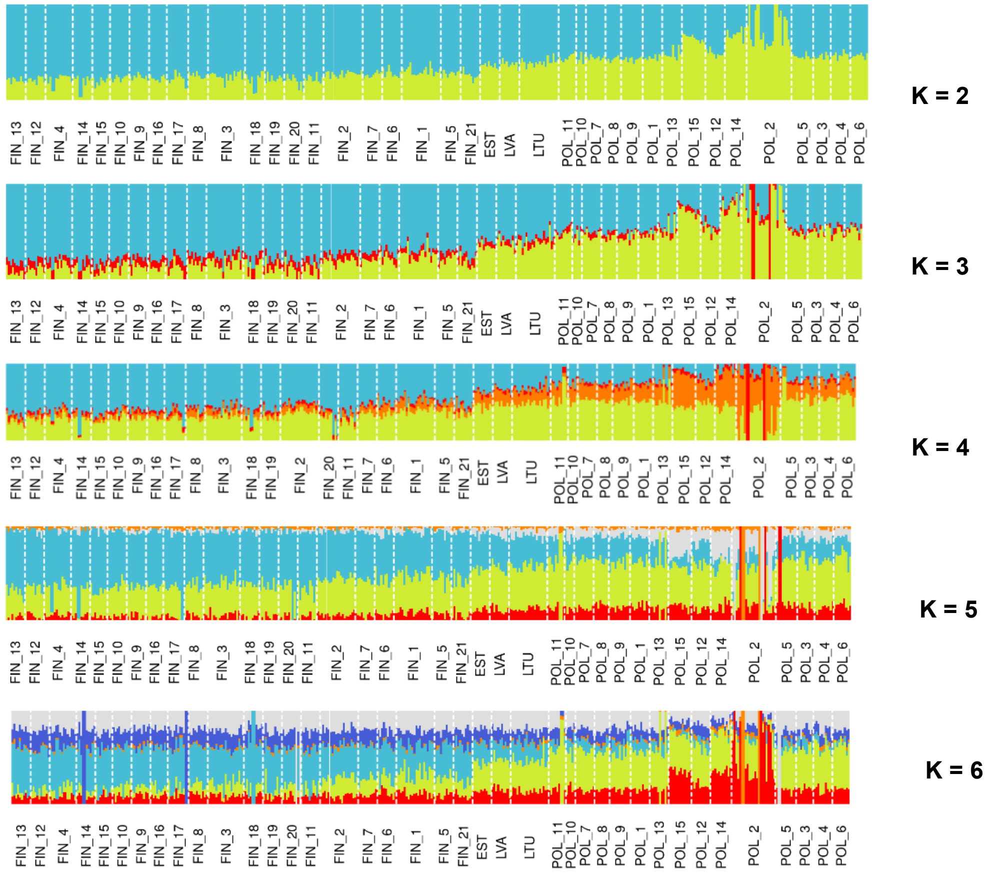

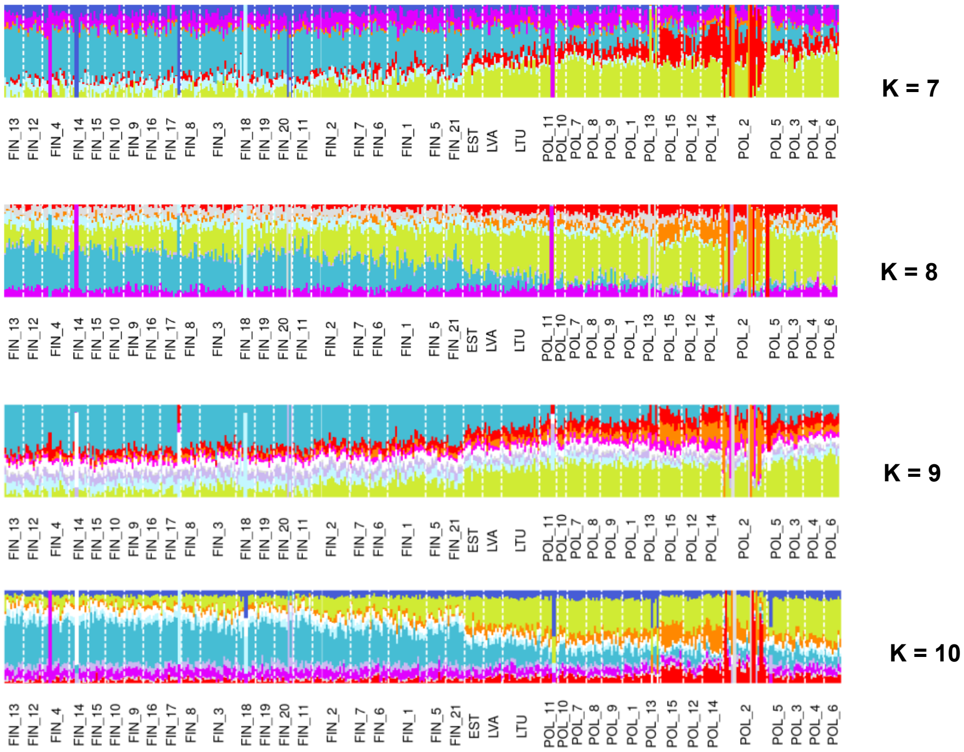
K = 7
K = 8
K = 9
K = 10
FIN_13
FIN_12
FIN_4
FIN_14
FIN_15
FIN_10
FIN_9
FIN_16
FIN_17
FIN_8
FIN_3
FIN_18
FIN_19
FIN_20
FIN_11
FIN_2
FIN_7
FIN_6
FIN_1
FIN_5
FIN_21
EST
LVA
LTU
POL_11
POL_10
POL_7
POL_8
POL_9
POL_1
POL_13
POL_15
POL_12
POL_14
POL_2
POL_5
POL_3
POL_4
POL_6

Figure S6. Principal component analysis (PCA) projections of *P. sylvestris* on a regional scale. (A) PCA for populations from Finland. Individuals are assigned to the northern and southern group, based on the ordination in environmental space as in Fig S1. (B) PCA for populations from Poland. Individuals from Stołowe Mountain. region were assigned to one group, and all the other populations were grouped together, based on the results of LEA analysis and ordination in environmental space in as Fig S1.

A

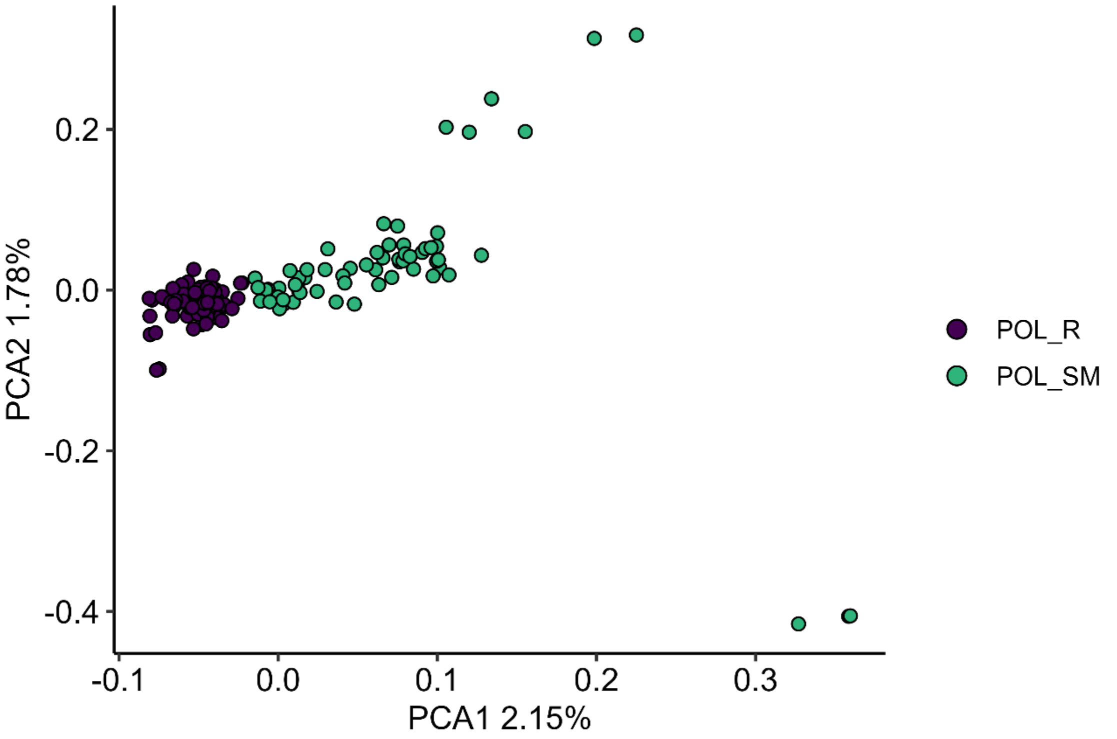

B

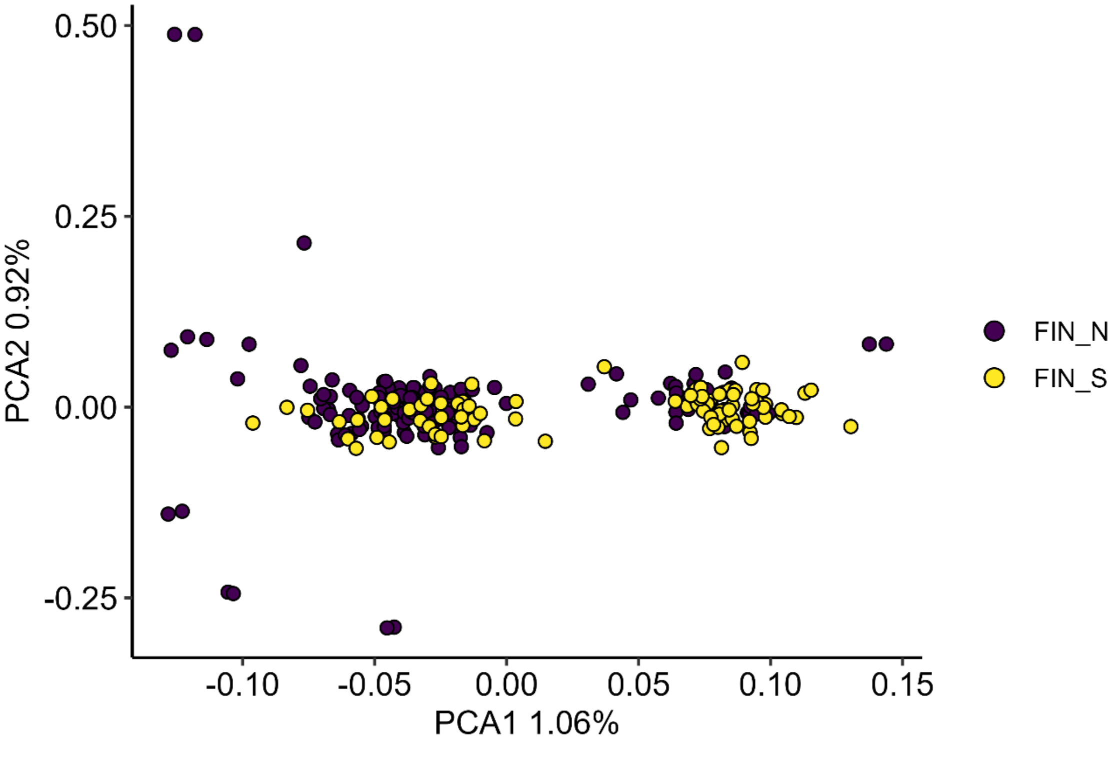

Figure S7. Comparison of PCA based on different numbers of SNPs included. (A) PCA based on all 440 samples and all SNPs after LD pruning (6995 SNPs - identical to the PCA in Fig. 2C). (B) PCA based on all 440 samples and putatively neutral SNPs (6975 SNPs, LD pruning, PAVs excluded). (C) PCA based only on PAVs (20 SNP, LD pruning) (D) Correlation between PC scores from PCA with all SNPs (6995) and with PAV SNPs only (20) with 1:1 identity line. Due to the PC scores being centered, this method can estimate how similar are two different PCA runs. The correlation was rather weak, but statistically significant, with $R^2 = 0.221$. We used permutation test based on random resampling of 20 SNPs from the whole dataset 10,000 times and calculated correlation for each resampling. The p value based on the permutation test was 0.0274, since randomly selected SNPs do not explain observed population structure.

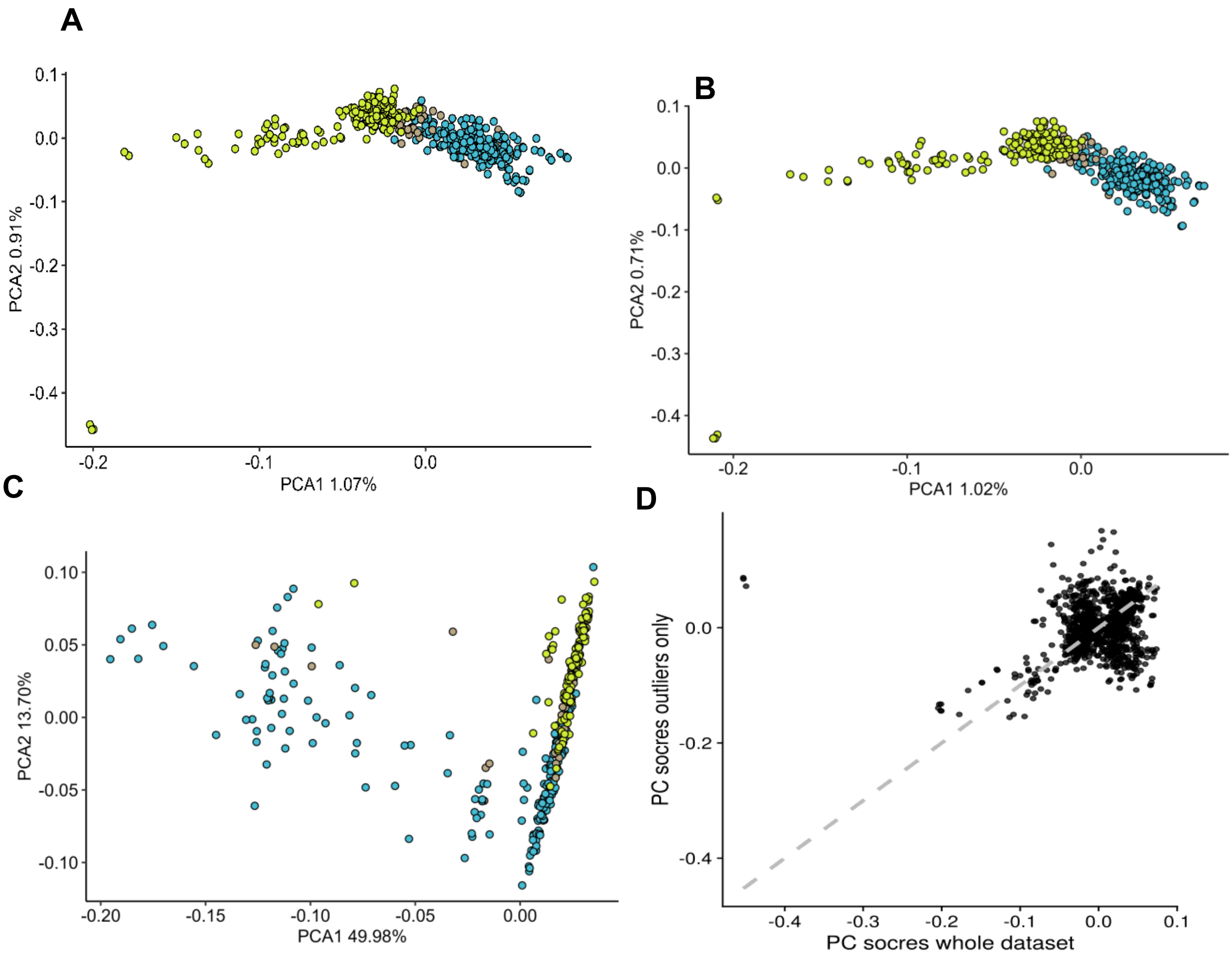

Figure S8. (A) Correlations between the environmental variables selected for redundancy analysis. (B) The results of RDA analysis on the ordination plot of the first two RDA axes. The points represent the populations colour coded as in Fig. 2, while the blue arrows represent the environmental predictors. The relative arrangement of the points and arrows on the plot represents their relationship to the RDA ordination axes. (C) Histograms of 55 SNPs detected by RDA and their correlations with the associated environmental variables. The vertical black line denotes a cutoff point of low correlation strength (none of the SNPs were found to be below that threshold); Abbreviations - meantemp: annual mean temperature; meandr: mean diurnal range; temps: temperature seasonality; percdrym: precipitation of driest month; percwetq: precipitation of wettest quarter; ph: top soil pH, wet: number of days receiving ≥0.1 mm precipitation; carbon: Organic carbon content. (D) Screeplot showing the variance explained by successive RDA axes. The first axis captured the majority of the constrained variance and was therefore the main focus for outlier detection.

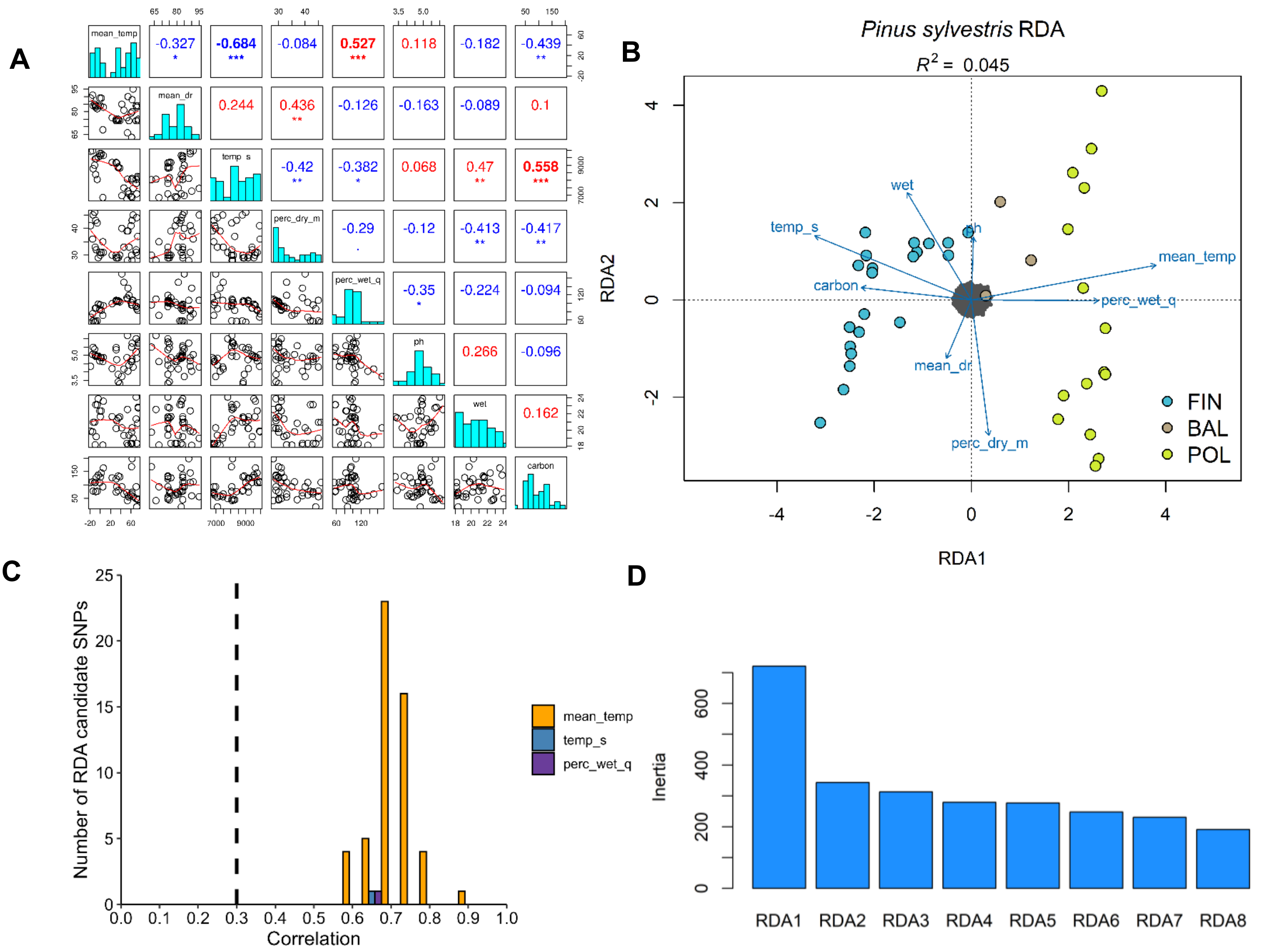

Figure S9. Correlation between genotype and mean annual temperature using a generalized linear model (GLM) with a binomial error for PAV SNPs. On the x-axis genotypes are coded as 0,1,2 – for homozygote, heterozygote and alternative homozygote; while on y-axis, temperature is in °C.

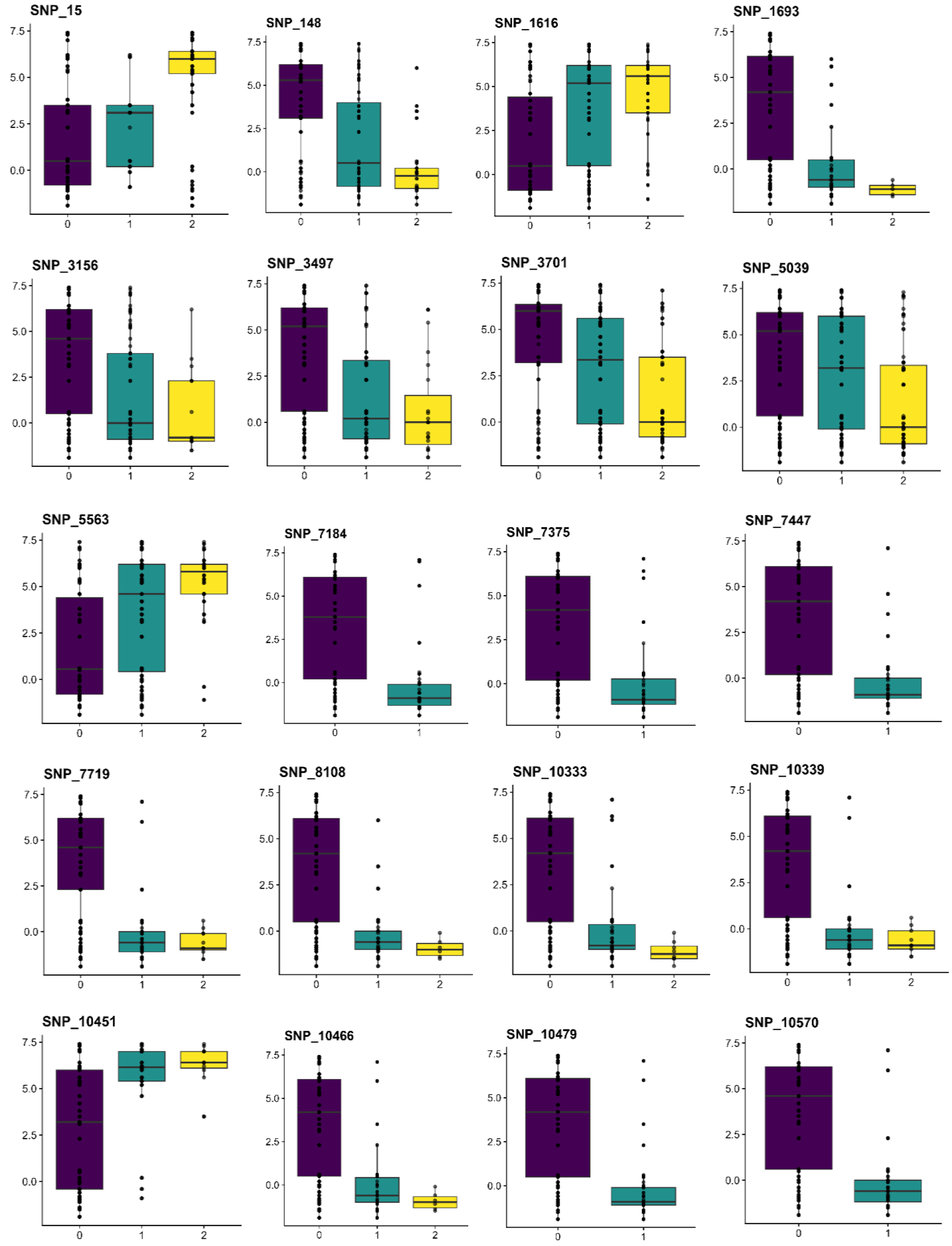

Figure S10. Venn diagram with the number of SNPs detected as outlier using only genomic outliers scans, separately in both regions. (A) Outliers SNPs detected in Finland. (B) Outliers SNPs detected in Poland. (C) Venn diagram with the number of SNPs concordant between two genomic outliers scans in comparisons between Poland (POL), Finland (FIN) and across whole studied transect (ALL).

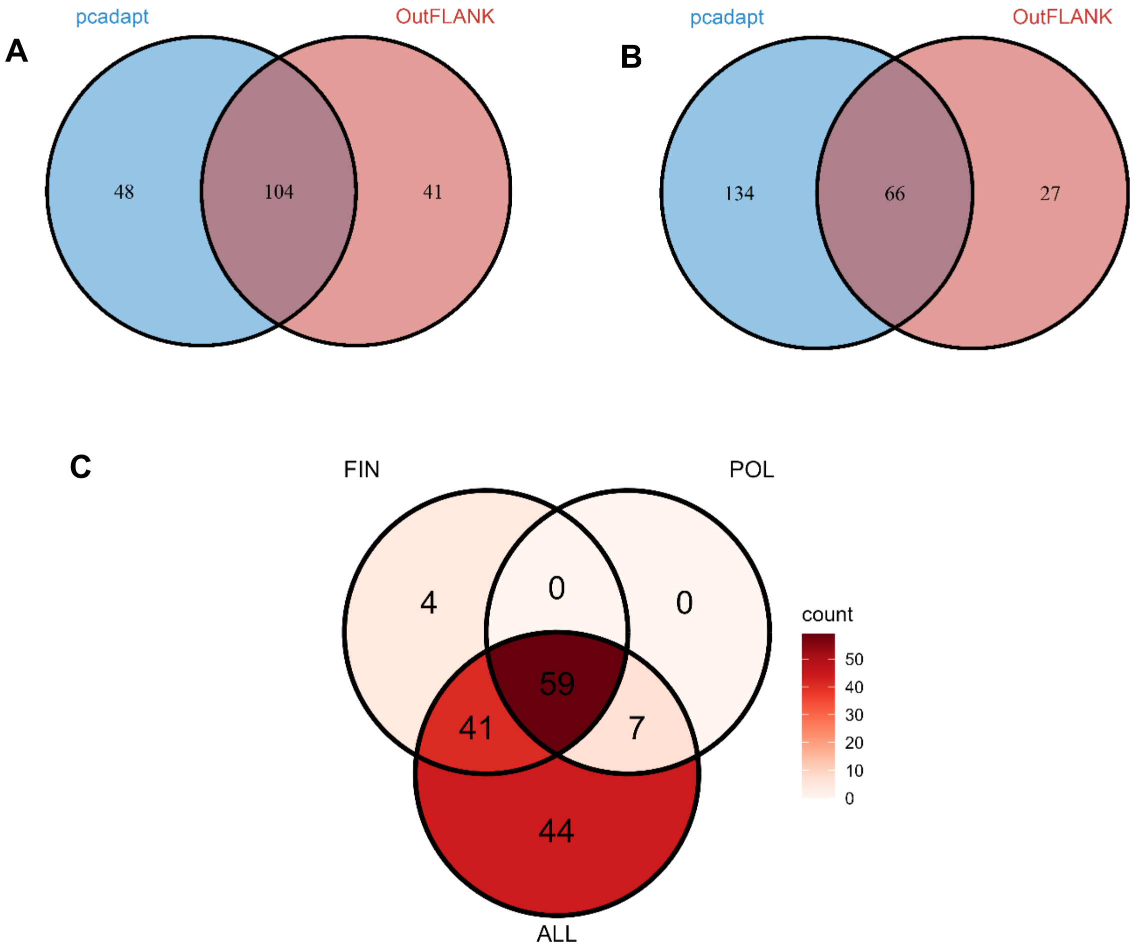

Figure S11. (A–C) Genomic offset under three future climate scenarios (SSP126, SSP245, SSP585) based on 20 potential adaptive variants (PAVs) identified across GEA methods. (A) The results of the genomic offset based on RONA-RDA methods. Genomic offset score is reflecting the mean amount of allelic shift based on all 20 PAVs. (B) Genomic offset projections based on RDA offset method, reflecting differences in adaptive index for current and future climate under three emissions scenarios. (C) Genomic offset projections based on gradient forest method, calculated as the multivariate environmental distance between present and future climates, weighted by the importance of allele–environment associations inferred from regression trees. (D) Correlations of genomic offset scores from RONA-RDA and RDA offset methods for three future climatic scenarios. (E) Correlations of genomic offset scores from RONA-RDA and GF offset methods for three future climatic scenarios.

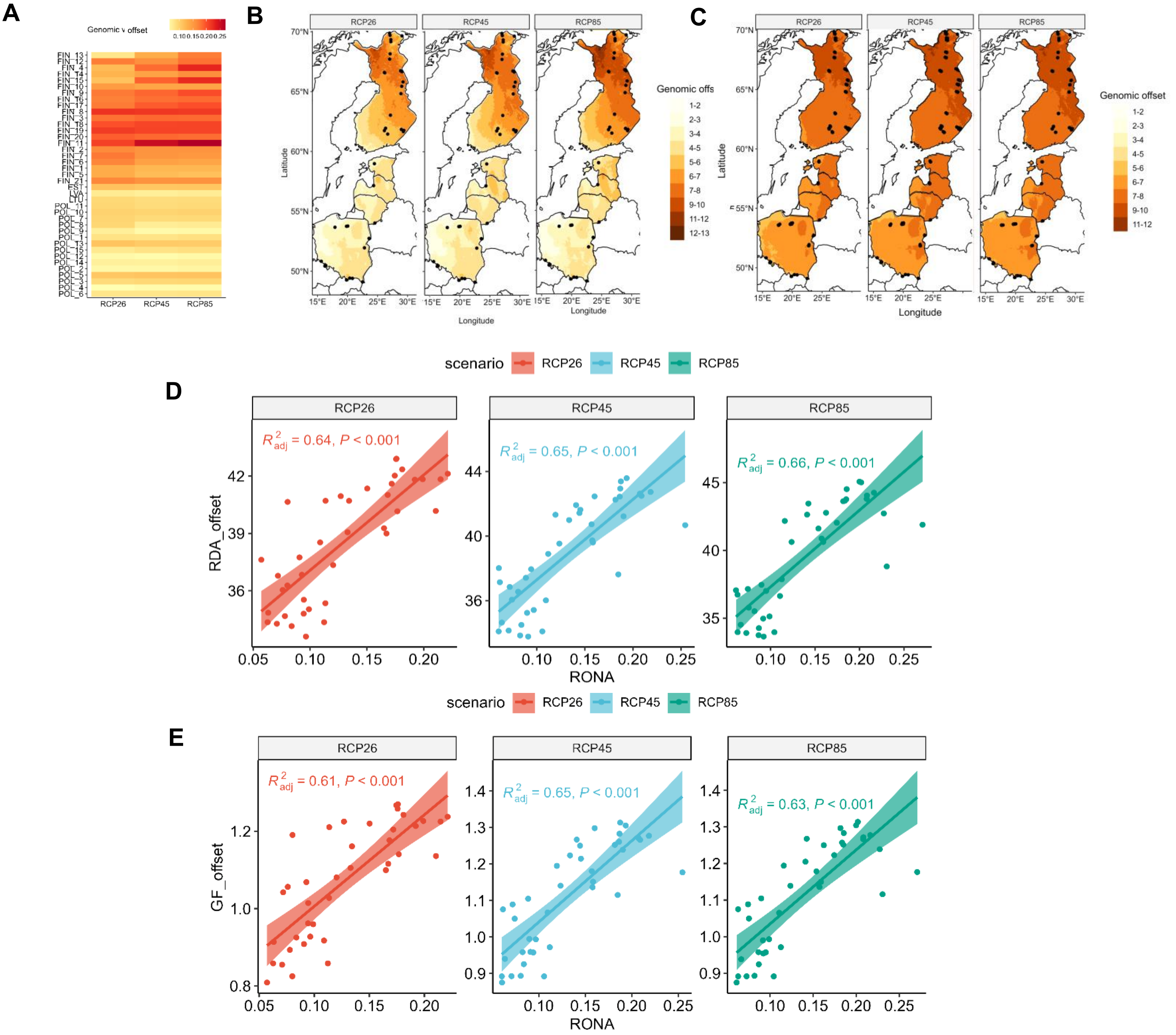

Figure S12. Genomic offset estimates under three climate scenarios (RCP2.6, RCP4.5, RCP8.5) using different methods and SNP panels. (A–C) Spatial projections of genomic offset calculated from the broader outlier panel (164 SNPs detected by at least two independent methods). Methods: (A) RONA-RDA, (B) RDA offset, (C) Gradient Forest. (D–F) Correlations between genomic offset values derived from the broader outlier panel (164 SNPs) and the conservative panel of 20 PAVs. Methods: (D) RONA-RDA, (E) RDA offset, (F) Gradient Forest.

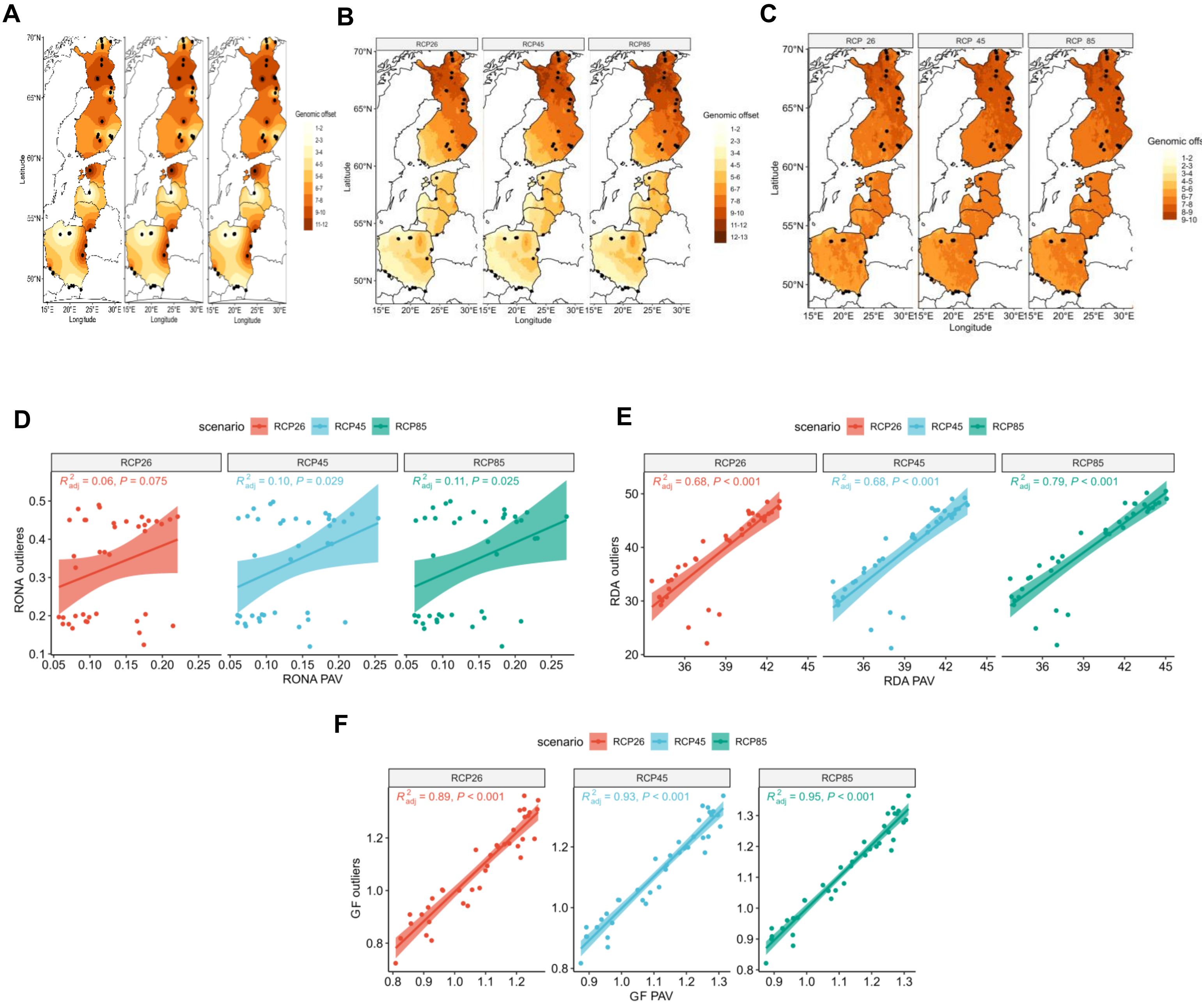

Figure S13. Number of generations required for the shift in allele frequency under selection starting from different initial allele frequencies. The frequency change equal to the minimum value (A) and maximum value (B) of genomic offset (0.06 and 0.27, respectively) was simulated under different selection coefficients (s in range 0.1-0.9) starting from different initial frequencies (0.01 – 0.9) with an increment of 0.01. Left: boxplot of the mean number of generations required for the shift at different values of s. Right – relationship between starting allele frequency and number of generations to shift allele frequency under different selection coefficients.

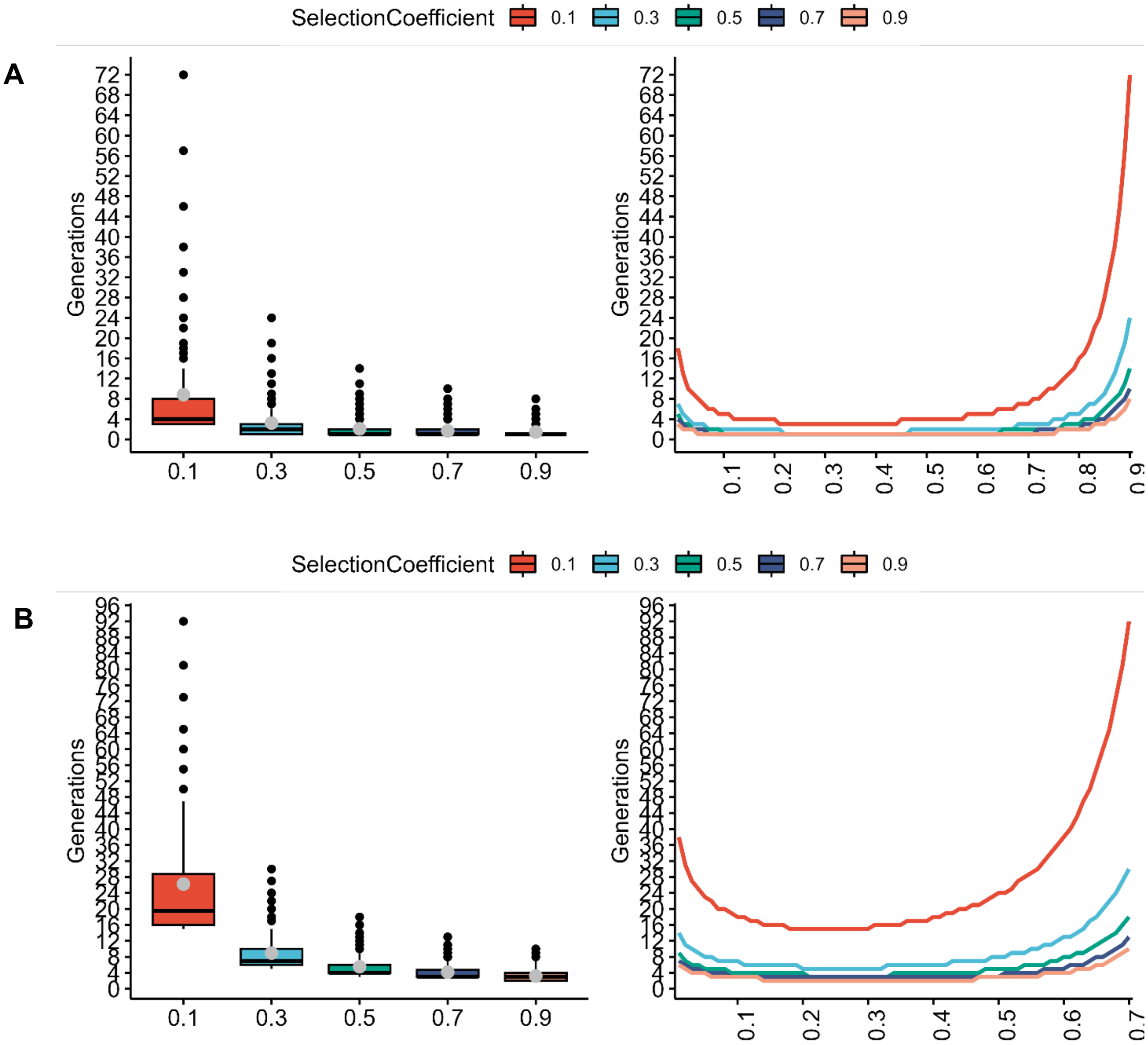

Supplement: Supplementary file 1 — Table S1: Location of the investigated populations of Pinus sylvestris . Table S2:. List of 25 environmental variables selected initially for RDA analysis. Bolded are the variables retained after checking multicollinearity. Table S3: Basic summary statistic for Pinus sylvestris populations in the studied transect. Table S4:. Results of the Generalized Linear Model (GLM) analysis assessing the association between potentially adaptive variants (PAVs) and temperature. The model was fitted using a binomial regression with a logit link function, where the log odds of genotype occurrence were modeled as a function of temperature. See Figure S9 for visual representation of those results with boxplots. Table S5:. Results of BLAST analysis of 20 PAV in Scots pine. The analysis was conducted using transcriptomic regions containing the focal single nucleotide polymorphisms (SNPs) as queries. For each SNP, the corresponding Axiom ID, transcriptomic region, BLAST accession number, and identified gene product are reported. Table S6:. Redundancy analysis (RDA) to partition among population genetic variation (F) in Pinus sylvestris into environment (env.), geography (geog.) and their combined effects, shown in the table as measured by adjusted R2. The proportions of the variation that were exclusively attributed to environment or geography are highlighted in light grey. The individual fractions of the variation that were confounded between various combinations of these two components are highlighted in dark grey. Table S7:. Genomic offset values for Scots pine populations under three climate change scenarios (SSP 126, SSP 245, and SSP 585), estimated using three approaches: (i) RONA‐RDA, which quantifies predicted offset as the mean allele‐frequency shift required under future climates; (ii) RDA offset, based on redundancy analysis; and (iii) Gradient Forest offset, which calculates the multivariate environmental distance between present and future climates weighted by the importan [file EVA-18-e70180-s001.pdf]
